# A Unifying Framework for Evaluating the Predictive Power of Genetic Variants Based on the Level of Heritability Explained

**DOI:** 10.1371/journal.pgen.1001230

**Published:** 2010-12-02

**Authors:** Hon-Cheong So, Pak C. Sham

**Affiliations:** 1 Department of Psychiatry, University of Hong Kong, Hong Kong, China; 2 Genome Research Centre, University of Hong Kong, Hong Kong, China; 3 State Key Laboratory of Brain and Cognitive Sciences, University of Hong Kong, Hong Kong, China; Queensland Institute of Medical Research, Australia

## Abstract

An increasing number of genetic variants have been identified for many complex diseases. However, it is controversial whether risk prediction based on genomic profiles will be useful clinically. Appropriate statistical measures to evaluate the performance of genetic risk prediction models are required. Previous studies have mainly focused on the use of the area under the receiver operating characteristic (ROC) curve, or AUC, to judge the predictive value of genetic tests. However, AUC has its limitations and should be complemented by other measures. In this study, we develop a novel unifying statistical framework that connects a large variety of predictive indices together. We showed that, given the overall disease probability and the level of variance in total liability (or heritability) explained by the genetic variants, we can estimate analytically a large variety of prediction metrics, for example the AUC, the mean risk difference between cases and non-cases, the net reclassification improvement (ability to reclassify people into high- and low-risk categories), the proportion of cases explained by a specific percentile of population at the highest risk, the variance of predicted risks, and the risk at any percentile. We also demonstrate how to construct graphs to visualize the performance of risk models, such as the ROC curve, the density of risks, and the predictiveness curve (disease risk plotted against risk percentile). The results from simulations match very well with our theoretical estimates. Finally we apply the methodology to nine complex diseases, evaluating the predictive power of genetic tests based on known susceptibility variants for each trait.

## Author Summary

Recently many genetic variants have been established for diseases, and the findings have raised hope for risk prediction based on genomic profiles. However, we need to have proper statistical measures to assess the usefulness of such tests. In this study, we developed a statistical framework which enables us to evaluate many predictive indices analytically. It is based on the liability threshold model, which postulates a latent liability that is normally distributed. Affected individuals are assumed to have a liability exceeding a certain threshold. We demonstrated that, given the overall disease probability and variance in liability explained by the genetic markers, we can compute a variety of predictive indices. An example is the area under the receiver operating characteristic (ROC) curve, or AUC, which is very commonly employed. However, the limitations of AUC are often ignored, and we proposed complementing it with other indices. We have therefore also computed other metrics like the average difference in risks between cases and non-cases, the ability of reclassification into high- and low-risk categories, and the proportion of cases accounted for by a certain percentile of population at the highest risk. We also derived how to construct graphs showing the risk distribution in population.

## Introduction

Genome-wide association studies have succeeded in uncovering many common genetic variants underlying complex diseases, raising hope for individualized risk prediction based on genomic profiles. Although the effect size of a single genetic marker is typically very modest and unlikely to be useful in risk prediction, prediction based on a collection of susceptibility variants may be promising. Several commercial companies (such as deCODEme, Navigenics, 23andMe) are already offering disease risk estimates based on genomic profiles of susceptibility variants. However, it remains controversial whether such tests will be useful clinically. This calls for appropriate measures to evaluate the performance of genetic risk prediction models. It should be noted that a high level of statistical significance does not equate to good predictive power [1], [2].

A very popular method of assessing the discriminatory ability of test is the AUC (also known as the c statistic). AUC can be defined as the area under the receiver operating curve, which is a plot of sensitivity versus 1-specificity. AUC is also equal to the probability that the test score or predicted probability is higher for a case than a non-case. A few previous studies have investigated the use and performance of AUC in genetic tests. Janssens et al. [3] performed a simulation study for the AUC achieved under different combinations of risk allele frequencies, odds ratios and disease prevalence. Lu and Elston [4] proposed an approach to construct the optimal ROC curve based on likelihood ratios. It has also been observed that the increase in AUC by SNPs to existing risk factors is in general modest [5].

Despite its widespread use, the ROC curve and AUC are not without limitations and they are not the only ways to assess the performance of a prediction model. For instance, it has been pointed out that that the AUC is not directly related to the absolute disease risks (i.e. probability of disease given test result), which is often of great clinical interest [6], [7]. Very large OR are often required to increase the AUC beyond existing risk factors [8]. In view of these limitations, other indices have been developed. These included the net reclassification improvement (NRI) and the integrated discrimination improvement [9]. The former is concerned with reclassification of subjects into risk categories and the latter with the mean risk difference in cases and non-cases. Graphs that display the risk distribution in the population have been advocated, for example the “predictiveness curve” [7].

In this study, we propose a novel unifying statistical framework that connects different measures of predictive power together. The framework is based on the liability threshold model, which assumes an underlying liability that is normally distributed. Affected individuals have a liability above the threshold. We show that given the overall disease prevalence and total variance in liability explained (equivalent to heritability explained) by the set of susceptibility variants, it is possible to estimate analytically the aforementioned prediction indices and construct graphs to visualize the performance of risk models.

## Methods

Here we establish links to various measures of predictive power within our variance explained framework. We will derive analytic expressions to evaluate different prediction indices.

### The liability threshold model

Our statistical framework is based on the liability threshold model. The methods to derive total variance in liability explained (V_m_) by known variants (or other risk factors) and the corresponding liability score for each genotype will be presented elsewhere (So et al., submitted [10]). It is useful to note this V_m_ can be interpreted as the total heritability attributed to the known variants. We partition the total liability into two components, one comprising known risk factors (i.e. “measurable” liability) and the other comprising other risk factors yet to be found. The measurable liability is normally distributed with mean 0 and variance equal to the total variance (or heritability) explained by the known variants (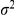). The overall liability conditioned on the measurable liability (*z*) is normally distributed with mean *z* and variance 1−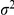. V_m_ and 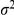 are used interchangeably in this paper, with the former usually referring to the concept of variance explained and the latter referring to its level.

### ROC curve and AUC

Consider a 2×2 table with disease status and test result (Table 1). Test is defined as positive if the level of measurable liability exceeds certain percentile cut-off, *c*. The absolute disease risk (*R*) is given by the chance that the overall liability, conditioned on the measurable liability, exceeds the threshold. *R* can be expressed as 
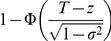
, where *T* is the liability threshold. The liability threshold is related to the overall probability of disease in the population (*K*), with 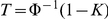. While we shall mainly focus on lifetime risks in this study, *K* can also be the probability of disease in a given period of time, say the 5-year or 10-year risk of disease. It is easy to see that the measurable liability is monotone increasing with the predicted risk, thus ROC curve constructed using either criterion will be identical.

**Table 1:** A 2×2 classification table.

	Disease +ve	Disease −ve	Total	
Test +ve	TP	FP	1-percentile	(*c*)
Test −ve	FN	TN	percentile	(1−*c*)
	*K*	1−*K*	1	

*K*, overall probability of disease in the population; TP: True positive; FP, false positive; FN, false negative; TN, true negative. *c*, percentile cut-off.

To construct the ROC curve, we need to evaluate specificity and sensitivity at different percentile cut-offs. Test positive can be defined as having a measured liability higher than a certain percentile cut-off *c*. Pr(Disease +ve|test+ve) (or the positive predictive value) is given by the average risk of people whose percentiles of measured liability exceeds the given cut-off *c*:
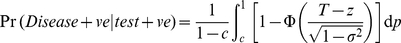
where *z* denotes the liability score at a certain percentile *from the distribution of measurable genetic factors*. *p* is a random variable representing the percentile of the measurable liability. Note that we need to integrate over all the percentiles above the cut-off *c*.

*z* can also be expressed as the inverse normal of *p*, i.e. 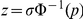. Hence we have
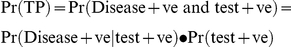


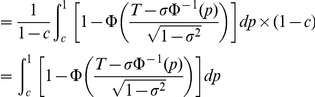
The other cells in the 2×2 table are computed readily given Pr(TP). Sensitivity and specificity are calculated using 5000 percentile cut-offs. Area under the curve (AUC) is then estimated from the graph using the R function integrate.xy from the sfsmisc package. Note that the positive and negative predictive values of the test can also be obtained for any arbitrary percentile cut-off.

### Approximation of TPR, FPR, and AUC by the binormal ROC curve

The binormal ROC curve is a classic example in ROC methodologies. In this model, we assume the test results are normally distributed in the affected and unaffected individuals. In our case, the test results are the measurable liability scores. We have already assumed the measurable liability follows a normal distribution *in the whole population*. The distributions of the measurable liabilities *conditioned on the affection status* are also usually close to normal curves. By the Pearson-Aitken (PA) formula [11], [12], we can estimate the mean and variance of the liability conditioned on the affection status and derive the AUC.

#### Mean measurable liabilities in affected and unaffected individuals

We consider the overall and measurable liability of an individual, denoted by 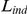 and 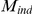 respectively. Selection for affected individuals changes the overall liability to a truncated normal distribution, with mean 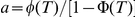 and variance 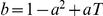. We shall use the subscripts *A* and 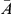 to denote affected and unaffected individuals respectively. Applying the Pearson-Aitken selection formula, it can be shown that within *affected* individuals, the mean of 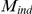 = 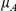 = *aσ* ^2^ and the variance of 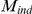 = 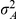 = *σ* ^2^ [1−(1−*b*) *σ* ^2^].

Similarly, selection for unaffected individuals changes the overall liability to a truncated normal distribution (this time the truncation is from above) with mean 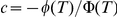 and variance *d* = 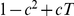. Within *unaffected* subjects, the mean of 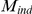 = 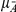 = *cσ* ^2^ and the variance of 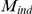 = 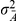 = *σ* ^2^[1−(1−*d*) *σ* ^2^].

#### The approximation formulas

The measurable liabilities in the affected and unaffected groups can be assumed to follow normal distributions:
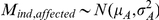
and
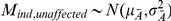
The AUC for the binormal ROC curve can be expressed in a simple form [13] :
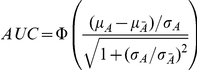
We may assume that the variances of measurable liability in the affected and unaffected groups are approximately equal, especially for more common diseases. AUC can then be approximated by the following formula (see supplementary methods in Text S1):
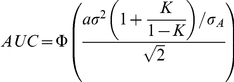
where 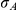 is the standard deviation in cases. This method of estimating AUC does not involve any numerical integration or simulations and can be easily implemented in a spreadsheet. Alternatively, to improve the accuracy of AUC estimate, we may calculate 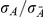 using the actual standard deviations of measurable liability (derived using the PA formula) in affected and unaffected groups. The formulas have been described previously.

By assuming normality in the measurable liability distributions in cases and controls, we can also provide simple formulas to approximate the TPR (i.e. sensitivity) and FPR (i.e. 1-specificity) given a risk threshold. The idea is to convert the absolute risk to its corresponding risk percentile within cases or controls. Within cases, we have
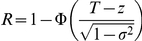
and
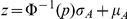
Substituting the 2nd expression into the 1st and change *p* to be the subject of formula,
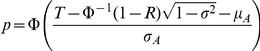
Within cases, a proportion of (1−*p*) of them have predicted risks above the risk threshold *R* and will be classified as “high-risk”. The true positive rate (TPR) is simply 1−*p*. By the same argument, we can also compute the approximate false positive rate (FPR) ( = 1- specificity), by
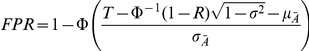



### Distribution of predicted risks given a set of known susceptibility variants

A major purpose of risk prediction is to stratify individuals into different risk categories of clinical importance. The ROC curve, however, does not typically display risk thresholds. As shown by Pepe et al. [7], although one can locate a point on the ROC curve corresponding to a given risk threshold, it is impossible to compare the population performances of different models using a particular risk criterion. This is because the point corresponding to the same threshold will be at different horizontal and vertical positions. Other measures and plots are more useful if clinically relevant risk thresholds or categories are concerned, such as plots of the predicted risk distribution in the population.

#### Risk distribution in the whole population

Suppose we are going to predict disease risks based on a set of known susceptibility genes or variants. What will be the distribution of the predicted risks in the whole population?

The predicted absolute risk of disease *R* can be represented by
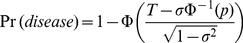
(1)where *T* is the liability threshold, 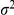 is the variance explained and *p* is the percentile of measurable risk derived from known genetic factors.

The graph of cumulative density function (cdf) of the predicted absolute risk can be obtained by plotting the risk percentiles against the predicted risks. The probability density function (pdf) [denoted *g*(*R*)] is derived by differentiation of the cdf, or *dp/dR*. The detailed mathematical derivations and results may be found in the supplementary methods (Text S1). We also derived formulas for the risk distribution within cases and controls.

#### The predictiveness curve

**Figure 1:**
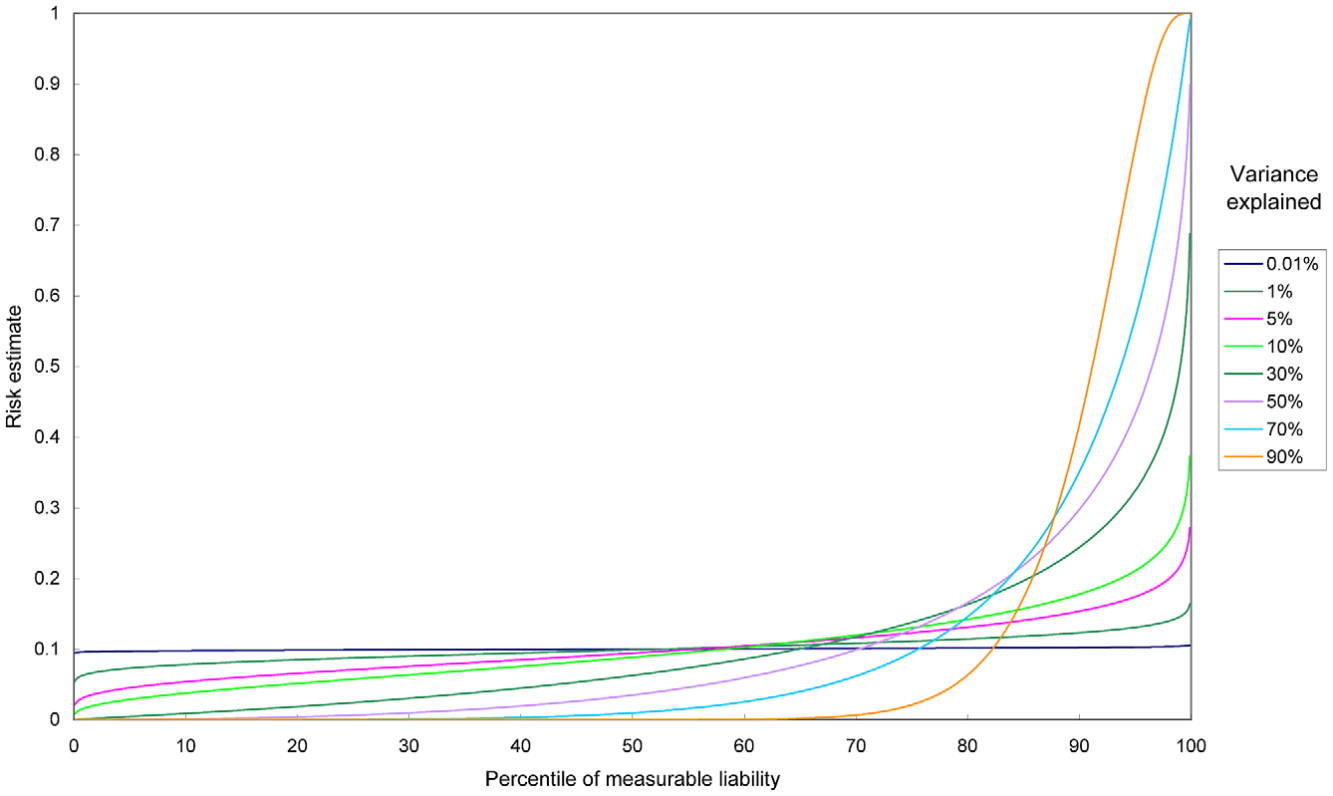
Predictiveness curves for different levels of variance explained. The percentile of measurable liability is equivalent to the percentile of absolute risk. The disease probability in the population is set at 0.1.

The predictiveness curve, first proposed by Pepe et al. [7], is a useful way to visualize the predictive power of a test and the distribution of risk in the population. This curve is constructed by plotting the predicted absolute disease risks against the percentiles of the absolute risks, or transposition of the cdf curve. Since the measurable liability has a monotonic relationship with the predicted absolute risk (i.e. a higher measurable liability always leads to a higher risk), the percentiles of the measurable liability can also be used. The curve may be produced based on equation (1), which relates the percentile and the predicted risk. Examples of predictiveness curves for different levels of variance explained are shown in Figure 1. This curve is useful for visualizing the proportion of population exceeding certain risk thresholds. Risk models can also be compared based on the same risk threshold. For example, adding susceptibility loci to a prediction model may increase the proportion of people exceeding the risk threshold. One may also look for the corresponding threshold given a percentile.

The predictiveness curve contains the same information as the pdf or cdf plots. The pdf curve is more intuitive, but it is also more difficult to derive and does not display the percentile and absolute risks directly.

### Area under the predictiveness curve and proportion of cases explained

One way to assess the predictive power is to estimate the proportion of cases that be accounted for by a given percentage (e.g. half) of the population at the highest risk. This measure, for instance, has been used to evaluate the predictive power of genetic factors for breast cancer [14]. It turns out that the area under the predictiveness curve is directly related to this measure.

**Figure 2:**
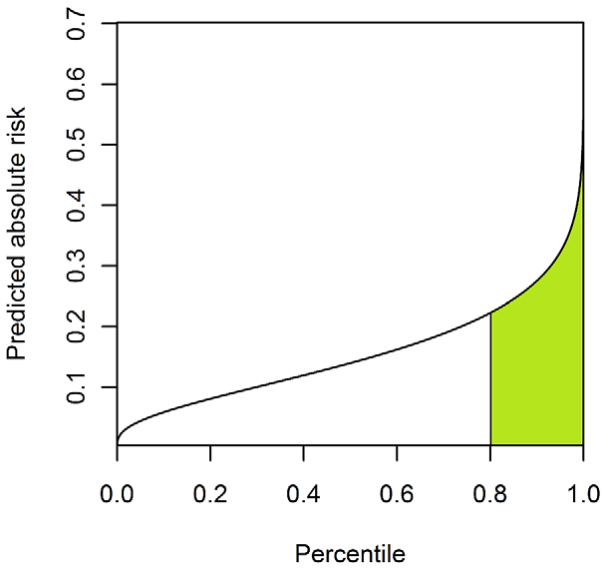
Area under the predictiveness curve and proportion of cases explained. The proportion of cases explained by the 20% population at the highest risk is equal to the green shaded area divided by the total area under the predictiveness curve.

The proportion of cases occurring in the Ptop% of the population at the highest risk is given by
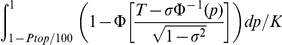
where *K* is the average probability of disease in the population. As an example, we consider the top 20% of the population at the highest risk in Figure 2. The proportion of cases explained by these 20% of population is equal to the green area divided by the total area under the predictiveness curve ( = *K*).

### Area under the curve when proportion of cases explained is plotted against population at the highest risk

We can plot the proportion of population at the highest risk (or equivalently the cumulative proportion of population arranged in *descending* order of their predicted risk) on the x-axis and the proportion of cases explained on the y-axis and obtained a curve. The area under this curve has been proposed as a measure of the AUC (area under the curve when sensitivity is plotted against 1-specificity). For example Clayton in a commentary [15] and a recent study in New England Journal of Medicine [16] consider the AUC of this plot to be equivalent to the AUC of the more conventional plot of sensitivity against (1-specificity). This is however *not* correct, as will be proved later. In fact, this curve may also be regarded as an alternative version of the Lorenz curve [17], originally used for showing wealth distribution. The Lorenz curve plots the cumulative proportion of population arranged in *ascending* order of their predicted risks on the x-axis and the cumulative proportion of cases on the y-axis. The area *above* the Lorenz curve is equivalent to the area *under* the curve when population at the highest risk is plotted against the proportion of cases explained. Note that similar to the ROC curve, the Lorenz curve does not contain information about the absolute risks.

We derive an analytic form of the conventional (or true) AUC and compare to the area under this new plot using the variance explained framework. We show that these two measures are not equal, but are close when the outcome is rare. Readers please refer to the supplementary methods (Text S1) for detailed derivations.

It can be shown that the true AUC is given by
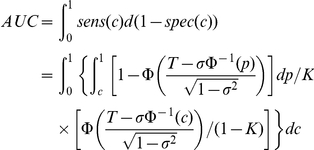
where *c* is the percentile cut-off of the measurable liability.

Now suppose we plot the proportion of population at the highest risk on the x-axis and the proportion of cases explained on the y-axis. The area under this curve is
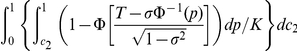
where *c_2_* is a percentile cut-off point.

This area differs from the previous (true) AUC by a factor of 
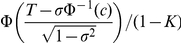
 in the integrand. When variance explained (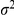) is small and *T* is large (i.e. the outcome is rare), the numerator is roughly equal to 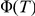, or (1−*K*). Thus the two measures are close when the outcome is rare, but are not identical mathematically.

### Net reclassification improvement (NRI)

For many diseases, there exist a priori risk categories to guide the plans of management for patients. For instance, the Third Adult Treatment Panel (ATP III) guideline suggests dividing the 10-year coronary heart disease risks into three categories (0 to 6, 6 to 20 and >20%). In clinical practice, we are often interested in whether adding a new biomarker or test will improve the re-classification of subjects into risk categories. The ability to reclassify subjects has therefore been suggested as a measure to assess a new biomarker or test [9]. This approach is not only clinically relevant, but may also be more sensitive than AUC, as shown in an example of predicting coronary heart disease by introducing high-density cholesterol to the model [9].

We now connect the variance explained framework to the notion of net reclassification improvement. Assume there are two prediction models which share all risk factors, except for a new biomarker. Our aim is to estimate the improvement in reclassification when the new marker is added. The performances of the models are considered *separately* for those who develop the event and for those who do not. We cross-tabulate the predicted absolute risks according to preset risk categories for the two models. An example of such a re-classification table can be found in Table 2 of Pencina et al [9].

**Table 2:** A typical re-classification table with 3 risk categories.

Old model (without the new biomarker)		New model (with the new biomarker)	
Predicted Risk	<6%	6–20%	>20%
Those who develop the event			
<6%	a_11_	a_12_	a_13_
6–20%	a_21_	a_22_	a_23_
>20%	a_31_	a_32_	a_33_
Those free of the event			
<6%	b_11_	b_12_	b_13_
6–20%	b_21_	b_22_	b_23_
>20%	b_31_	b_32_	b_33_

Let *D* be the event indicator. We classify downward movement (down) as a change to a lower risk category under the new model and upward movement (up) as a change to a higher risk category. Pencinia et al.[9] defined the net reclassification improvement (NRI) as
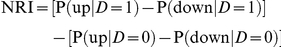
To evaluate the NRI under a variance explained framework, we need to consider three liability distributions: (1) the overall liability (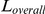), (2) the measurable liability under the old model (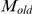) and (3) the measurable liability under the new model (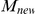). Note that 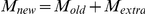, where 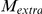 is the measurable liability attributed to the new risk factor(s) introduced. We assume the new risk factor is independent of the existing risk factors, or 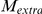 is independent of 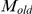. This may not be true in some cases, such as adding genetic markers associated with lipid levels or blood pressure to a prediction model of coronary artery disease that has already included these risk factors. The framework presented below can deal with such complications theoretically, however the covariance between 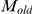 and 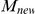 needs to be correctly specified beforehand.

The vector [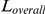, 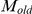, 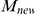] has the following mean and covariance matrices
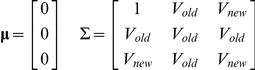
where *V*_old_ and *V*_new_ are the total variance explained under the old and new prediction models respectively.

Let us consider the group who develop the event of interest. Applying PA formula, the mean and variance of the vector [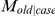, 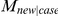] are as follows:
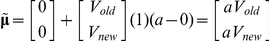


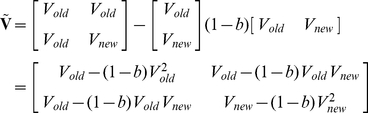
The mean and variance of 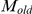 and 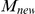 within the non-cases are calculated in a similar manner.

Recall that the predicted risk *R* can be calculated by
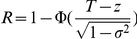
Hence
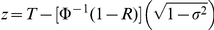
Suppose there are 3 preset risk categories: 0 to r_1_, r_1_ to r_2_ and r_2_ to 1. Note that the method can also be readily extended to deal with any number of categories. The corresponding quantile of the measurable liability (*z*) can readily be obtained from the above formula, given the variance explained. As an example, among the cases, the probability of moving from 0 to r_1_ category under the old model to the r_1_ to r_2_ category under the new model is given by
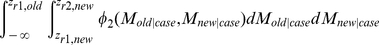
where 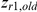, 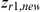 and 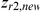 are the quantiles of the measurable liability corresponding to the specified risk under the old or new models. 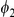 denotes the bivariate normal density function. Since the mean and covariance matrix of [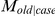, 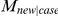] is known, the above integral can be readily computed. The probabilities of other patterns of changes in risk categories can be obtained by altering the upper and lower limits of the integral.

To illustrate how the NRI is calculated, consider the following example in which two models are used to predict the risk of an event and the risk categories are 0–6%, 6–20% and >20% (i.e. r_1_ = 0.06 and r_2_ = 0.2). Table 2 illustrates how the re-classification table would look like. Note that all table cells are expressed in probabilities, such that 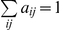 and 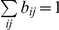. For instance, a_12_ = (no. of cases who moved from <6% category to 6–20% category) / (total no. of cases)

All the cells can be computed analytically by bivariate integration. For example, the bivariate integral shown above calculates cell a_12_. We have
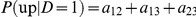


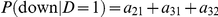


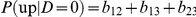


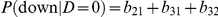



### Integrated discrimination improvement

The reclassification improvement is dependent on the choice of risk categories. Pencina et al. [9] suggests an extension of NRI to overcome this drawback. Instead of setting only a few categories, now each person represents a separate category. In this case, it is sensible to consider the actual changes in predicted probabilities rather than simply the directions of movement in risk categories. Denoting the predicted disease probabilities under the old and the new modes be 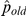 and 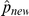 respectively, we have the following index known as integrated discrimination improvement:
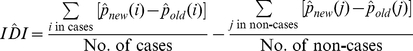
Pencina et al. showed that the above index has another intuitive meaning: it is equal to the difference in Yate's discrimination slopes (DS) [18] under the two models. The discrimination slope is equal to the difference in mean predicted risks in cases and non-cases. Therefore we have
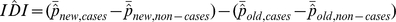
Since we have derived the pdf of predicted risks in case and non-cases previously, the discrimination slope can be deduced for any model and IDI can be calculated easily for two nested models. Let 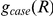 and 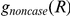 be the pdf of predicted risks in cases and non-cases respectively, the discrimination slope under a certain model is simply
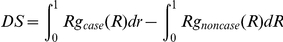



### Standard deviation of the predicted risks

The variance or standard deviation (SD) of the risk distribution is another index to the predictive ability of a test. A test which high discriminatory power should assign more dispersed risk estimates to subjects. In contrast, subjects' risk estimates tend to be close for a test that discriminates poorly.

If *g*(*R*) is the pdf of the risk distribution, the variance of the risk distribution is
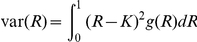
where *K* mean disease risk in the population. It is also useful to compare var(*R*) with the maximum variance that can be achieved. In the perfect case, all cases are assigned a risk of 1 and all non-cases are assigned a risk of 0. The variance of risk under this perfect scenario is given by the variance of a Bernoulli variable i.e. *K*(1−*K*). The ratio of the observed variance to the maximum achievable variance is hence
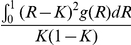
Interestingly, this ratio is equal to the mean risk difference between cases and controls, or the discrimination slope, as shown in Pepe *et al* [19]. The results in Table 3 confirmed this relationship.

**Table 3:** Predictive indices under different combinations of overall disease probability (*K*) and variance explained (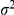).

	*K* = 0.005	*K* = 0.005	*K* = 0.005	*K* = 0.01	*K* = 0.01	*K* = 0.01	*K* = 0.05	*K* = 0.05	*K* = 0.05	*K* = 0.1	*K* = 0.1	*K* = 0.1
	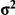 = 0.05	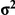 = 0.1	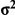 = 0.2	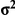 = 0.05	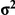 = 0.1	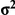 = 0.2	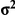 = 0.05	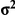 = 0.1	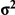 = 0.2	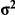 = 0.05	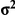 = 0.1	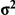 = 0.2
AUC accurate	0.678	0.746	0.832	0.666	0.730	0.814	0.635	0.690	0.765	0.622	0.672	0.742
AUC approx	0.677	0.742	0.821	0.665	0.726	0.803	0.634	0.686	0.754	0.621	0.669	0.731
AUC approx2	0.679	0.747	0.833	0.667	0.731	0.815	0.636	0.691	0.766	0.623	0.673	0.744
Prop cases exp 0.1	0.258	0.350	0.505	0.241	0.323	0.460	0.201	0.255	0.346	0.182	0.224	0.293
Prop cases exp 0.2	0.421	0.530	0.691	0.401	0.500	0.650	0.349	0.421	0.535	0.323	0.382	0.474
Prop cases exp 0.5	0.746	0.831	0.924	0.729	0.812	0.906	0.681	0.752	0.845	0.656	0.719	0.805
Var of risk	1.23E-05	2.90E-05	7.97E-05	4.06E-05	9.27E-05	2.39E-04	5.68E-04	1.21E-03	2.75E-03	1.60E-03	3.34E-03	7.20E-03
Var of risk to max	0.0025	0.0058	0.0160	0.0041	0.0094	0.0241	0.0120	0.0255	0.0578	0.0178	0.0371	0.0800
Mean risk in cases	0.0075	0.0108	0.0209	0.0141	0.0193	0.0339	0.0614	0.0743	0.1049	0.1160	0.1334	0.1720
Mean risk in noncases	0.0050	0.0050	0.0049	0.0100	0.0099	0.0098	0.0494	0.0487	0.0472	0.0982	0.0963	0.0921
Mean Risk Diff	0.0025	0.0058	0.0160	0.0041	0.0094	0.0241	0.0120	0.0255	0.0577	0.0178	0.0371	0.0799
Risk at 10th percentile	0.0017	0.0008	0.0002	0.0037	0.0020	0.0006	0.0238	0.0153	0.0066	0.0538	0.0377	0.0191
Risk at 90th percentile	0.0094	0.0111	0.0126	0.0182	0.0214	0.0250	0.0817	0.0957	0.1154	0.1537	0.1778	0.2142
RR from 10th to 90th	5.68	13.21	58.42	4.95	10.76	42.06	3.44	6.23	17.56	2.85	4.72	11.24
Range from 10th to 90th	0.0078	0.0102	0.0124	0.0145	0.0194	0.0244	0.0580	0.0803	0.1088	0.0998	0.1401	0.1951

AUC accurate, accurate AUC obtained by the integration formula as described in text; AUC approx, AUC approximated by binormal curve and assuming equal variance in cases and controls; AUC approx2, AUC approximated by binormal curve and *un*equal variance in cases and controls.

Prop cases exp 0.1, proportion of cases explained by the people at top 10% of risk; similar abbreviations used for the next 2 items.

Var : variance ; max, maximum ; Diff, difference; RR, relative risk.

### Simulations

We performed a simulation study to check our results. We simulated a hypothetical cohort study of 1 million people. The “true” predicted risk of disease is based on a logistic model,
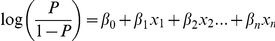
and
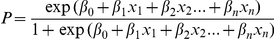
where *P* is the true predicted risk of disease. The outcomes of individuals were simulated from *P*. The outcome was set to 1 if a randomly generated uniform variable was less than the true predicted risk and vice versa. To ensure that the allele frequencies and effect sizes in our simulations are realistic, we extracted the allele frequencies and odds ratios from a list of 30 known susceptibility variants for prostate cancer. The data were based on the National Human Genome Research Institute (NHGRI) catalog. The loci were assumed to be independent and genotypes (coded 0, 1 and 2) were simulated randomly from the allele frequencies. The model was additive on the log-odds scale, or equivalently multiplicative on the odds ratios scale. The regression coefficients 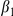
…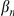 were derived from log(OR) and 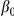 was adjusted to yield disease probabilities that were approximately equal to 0.005, 0.01 and 0.1. The precise disease probability is not important as our aim is just to compare theoretical and simulation results. We computed predictive metrics and constructed histograms of predicted risks and predictiveness curves based on the simulated disease status and predicted risks. The calculations were repeated using the allele frequencies and effect sizes of the first 10, 20 and 30 variants from Table S1. The somers2 function in the Hmisc package was used to compute the AUC.

To compute the NRI in simulations, the outcomes and true predicted risk were first simulated with the above method using all 30 loci. Then we fitted a logistic regression model using only the first 20 loci, treating it as the old model. The NRI from the old model (20 loci) to the new model (30 loci) was computed.

As a more technical consideration, the OR is not exactly equal to the risk ratio which is required for calculating the variance explained. Zhang and Yu [20] have proposed a simple correction of OR to the risk ratio in cohort studies. We modified their approach to accommodate the three genotypes (or “exposures”) in our case (So et al. submitted [10]). The resulting risk ratio estimates were used as inputs.

## Results

**Figure 3:**
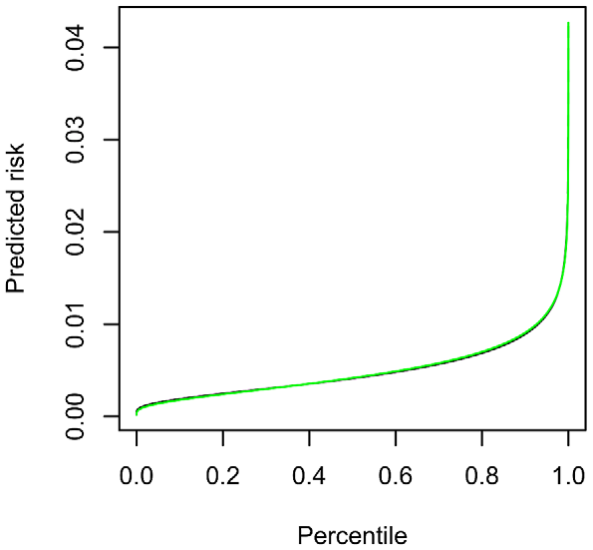
Comparison of the predictiveness curves from simulated data and theoretical calculations. The predictiveness curve plots the predicted risk against risk precentiles. The overall disease risk *K* = 0.005 and 30 loci from Table S1 were included. The black dotted line represents results from simulations and the green solid line is obtained by theoretical calculations. The total variance explained is equal to 0.0442. The theoretical estimates of predicted risks are from equation (1), i.e. 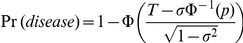, where *T* is the liability threshold, 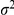 is the variance explained and *p* is the percentile of measurable risk derived from known genetic factors.

**Figure 4:**
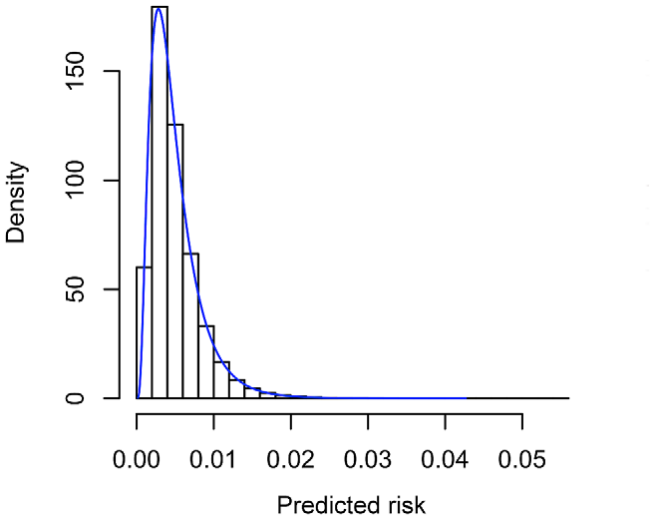
Comparison of predicted risk distributions from simulated data and theoretical calculations. The overall disease risk *K* = 0.005 and 30 loci from Table S1 were included. The histogram was obtained from simulations data, while the blue line showing probability density was derived from theoretical calculations. The theoretical distribution was obtained by differentiating the cumulative density function of estimated risks.

Predictive indices obtained by analytic calculations and simulations are presented in Table S2. The results from both approaches were very close in all scenarios. Figure 3 and Figure 4 show comparisons of the predictiveness curves and the risk distributions from simulations and our theoretical calculations. Again the graphs derived from analytic means match very well with the simulation results. The results confirm our derivations and show that a multiplicative model on the OR scale is very similar to an additive model on the liability scale. This is equivalent to the similarity between probit and logit models in generalized linear modeling [21]. Also of note is that the AUCs from the approximation formulas are close to the more accurate version. In addition, the AUCs are almost identical given an identical set of susceptibility SNPs, regardless of disease prevalence. This is because AUC only depends on the ranks of predicted risks but not actual risk levels. Table S3 shows the NRI and its components (probability of moving up or down categories in cases and non-cases) from theoretical derivations and simulations. They are reasonably close, though the discrepancy is slightly larger than in the previous table. The discrepancy may be due to difference in model assumptions (probit Vs logit) and slight sampling variability. As NRI involves the risks estimates from two models and their covariance, any difference in model assumptions may be exaggerated. NRI also involves the discretization of risks into categories in two models, and this may also lead to a slightly higher discrepancy.

We created some combinations of disease probability (*K*) and variance explained and computed the predictive metrics in each case (Table 3). As expected, all the prediction metrics improve when *V_m_* increases. This includes increases in AUC, proportion of cases explained by people at highest risk, variance of the predicted risk, the mean risk difference and the range and relative risk at the 10th and 90th percentiles.

We now considered how the predictive metrics changes at different prevalences, given a fixed level of V_m_. As shown in Table 3, the AUC and the proportion of cases explained decreases with increasing prevalence. The mean risk difference in cases and controls is wider when the disease is more common, given the same V_m_. The relative risk by comparing the risks at 10th and 90th percentiles is larger at lower prevalences, but the range is larger at higher prevalences.

Next some combinations of V_m_ under the old and new prediction models are considered (Table 4). The risk thresholds were set at 6% and 20% under overall prevalences of 5% and 10%. Under the simulated scenarios, we observed that the magnitude of NRI is the largest, followed by the increase in AUC, and the IDI is the smallest. It is noteworthy that it is possible the AUC increase is unimpressive but the NRI is more substantial. For example when the variance explained increases from 20 to 25% at *K* = 0.05 or 0.1, the AUC increases by only about 0.03 while the NRI is around 11%.

**Table 4:** Improvement in predictive indices with increase in variance explained.

*K*	*V_old_*	*V_new_*	NRI	AUC increase	IDI
0.05	0.05	0.1	0.099	0.055	0.014
	0.05	0.15	0.195	0.096	0.029
	0.1	0.15	0.102	0.041	0.015
	0.1	0.2	0.202	0.075	0.032
	0.2	0.25	0.104	0.029	0.019
	0.2	0.3	0.201	0.054	0.040
0.1	0.05	0.1	0.166	0.050	0.019
	0.05	0.15	0.306	0.089	0.040
	0.1	0.15	0.142	0.038	0.021
	0.1	0.2	0.262	0.070	0.043
	0.2	0.25	0.109	0.028	0.024
	0.2	0.3	0.205	0.053	0.049

*V_old_* and *V_new_* refers to the variance explained under the old and new models respectively.

Finally we applied our methodologies to a number of complex diseases to evaluate the predictive performance achievable by the known susceptibility loci (Table 5). To our knowledge, this is by far the most comprehensive assessment of predictive power based on established susceptibility variants, in terms of both the number of diseases and the variety of predictive metrics covered. We computed the total variance explained by established susceptibility variants for each disease. Details of the survey are provided elsewhere (So et al., submitted [10]).

**Table 5:** Predictive indices for nine complex diseases.

	Bipolar	Ca Breast	CAD	Crohn	Ca Prostate	SCZ	SLE	DM1	DM2
Lifetime Risk	0.021	0.127	0.3365	0.0060	0.156	0.0072	0.0031	0.0066	0.2895
*V_m_*	0.0214	0.057	0.123	0.074	0.125	0.003	0.087	0.109	0.118
No. of variants	5	13	12	32	27	4	23	45	25
AUC accurate	0.600	0.625	0.662	0.711	0.680	0.543	0.741	0.750	0.661
AUC approx	0.600	0.624	0.658	0.708	0.675	0.544	0.738	0.745	0.657
AUC approx2	0.601	0.626	0.664	0.712	0.681	0.544	0.742	0.751	0.663
Prop cases exp 0.1	0.174	0.181	0.173	0.298	0.218	0.130	0.345	0.353	0.180
Prop cases exp 0.2	0.310	0.322	0.314	0.471	0.376	0.246	0.523	0.535	0.324
Prop cases exp 0.5	0.639	0.656	0.655	0.788	0.716	0.562	0.825	0.835	0.664
Var of risk	5.68E-05	2.56E-03	1.66E-02	2.66E-05	7.62E-03	1.28E-06	1.05E-05	5.24E-05	1.41E-02
Var of risk to max	0.0028	0.0231	0.0745	0.0045	0.0578	0.0002	0.0034	0.0079	0.0687
Mean risk in cases	0.0237	0.1472	0.3859	0.0104	0.2048	0.0074	0.0065	0.0145	0.3383
Mean risk in noncases	0.0209	0.1241	0.3114	0.0059	0.1470	0.0072	0.0031	0.0066	0.2696
Mean Risk Diff	0.0028	0.0231	0.0745	0.0045	0.0578	0.0002	0.0034	0.0079	0.0687
Risk at 10th percentile	0.0124	0.0682	0.1759	0.0015	0.0588	0.0058	0.0005	0.0011	0.1444
Risk at 90th percentile	0.0310	0.1950	0.5119	0.0122	0.2754	0.0087	0.0067	0.0148	0.4517
RR from 10th to 90th	2.50	2.86	2.91	8.32	4.69	1.49	12.23	13.92	3.13
Range from 10th to 90th	0.019	0.127	0.336	0.011	0.217	0.003	0.006	0.014	0.307

*V_m_* , level of variance explained. Bipolar, bipolar disorder; Ca Breast, breast cancer; CAD, coronary artery disease; Crohn, Crohn's disease; Ca Prostate, prostate cancer; SCZ, schizophrenia; SLE, systemic lupus erythematosus; DM1, type 1 diabetes mellitus; DM2, type 2 diabetes mellitus.

**Figure 5:**
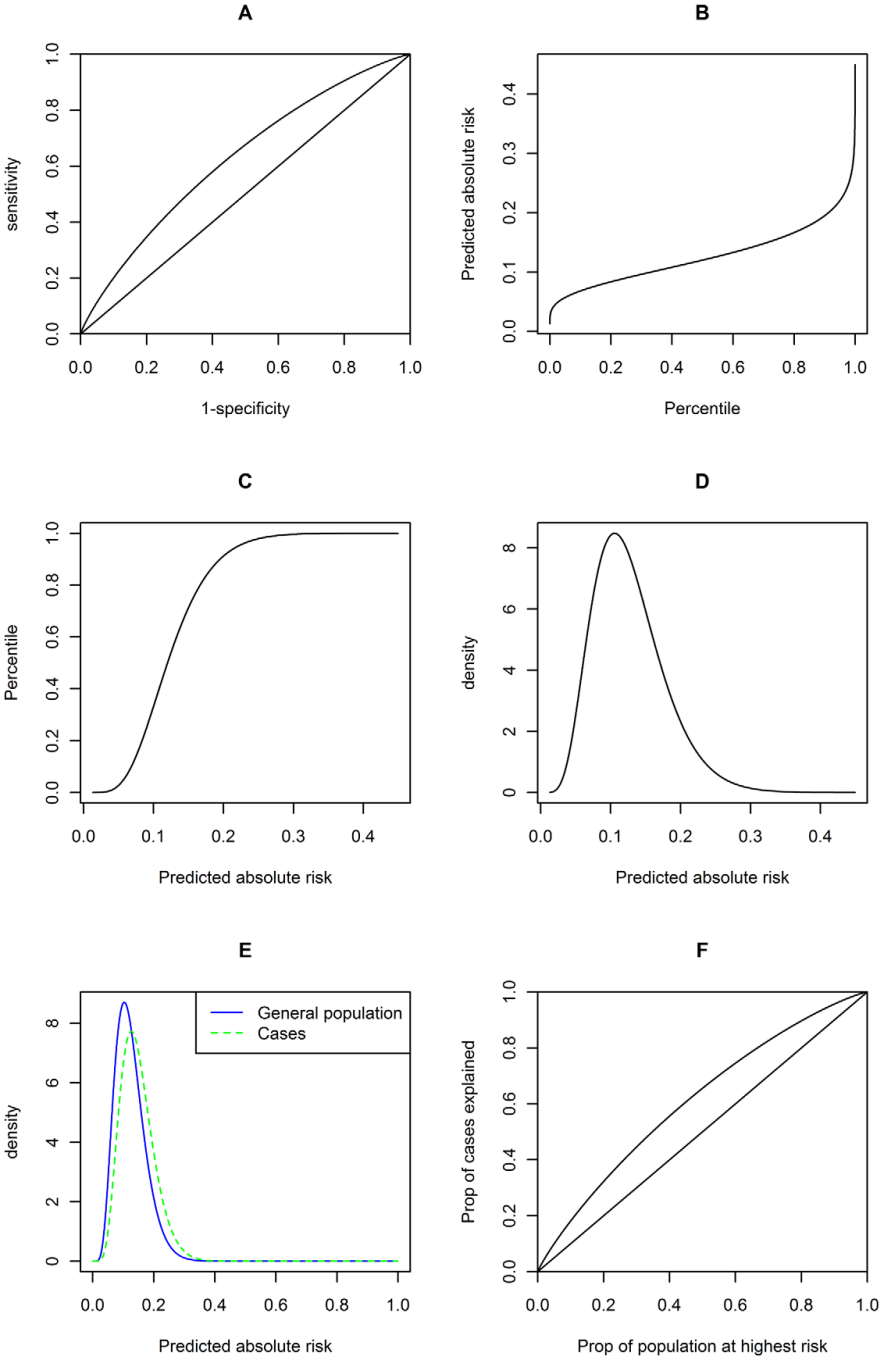
Graphs showing risk distribution and predictive power of known susceptibility variants for breast cancer. A: ROC curve ; B: predictiveness curve (predicted risk against risk perecentile) ; C: Cumulative density function of predicted risks ; D: Probability density function of predicted risks ; E: Probability density function of predicted risks in the population (blue solid line) and in cases (green dotted line) ; F: Proportion of cases explained against proportion of population at highest risk. While the plots A and F appear similar, they are not identical as shown mathematically in the text. Similar graphs for other diseases are presented in Figures S1, S2, S3, S4, S5, S6, S7, S8.

As shown in Table 5, the AUC for the diseases are in general not very high, but the AUC for type 1 diabetes and systemic lupus erythematosus (SLE) reach around 0.75, a threshold that may be considered clinically useful in discrimination [22]. (Also note that the MHC variants were not included for type 1 diabetes.) The mean risk differences in affected and unaffected individuals range from almost 0 to about 7%, the difference being larger for more common diseases. The relative risk and the range between the 10th and 90th percentiles were also shown. The largest relative risk was about 14 times (type 1 diabetes) and the widest range was around 30% (type 2 diabetes). We note that this sort of comparison may be quite arbitrary as one can compare any 2 percentile cut-offs. We merely aim at providing an idea of how dispersed the risks are at different percentiles. For a more comprehensive assessment, one can look at the predictiveness curve and use formulas derived before for the predicted risk at any percentile. Figure 5 contains a panel of six graphs showing the predictive performance of known variants for breast cancer. Similar graphs for the other diseases studied are shown in Figures S1, S2, S3, S4, S5, S6, S7, S8.

## Discussion

We have shown in this study that the variance explained framework enables us to evaluate the predictive or discriminatory power of tests analytically. Under this framework, the overall probability of disease and the total variance explained are the two fundamental quantities that determine the distribution of predicted risks and the predictive power. Note that we mainly considered lifetime risk in the current study, although one can also consider the probability of disease in a specified period of time. The distribution of predicted risks in the population can be derived analytically given the overall probability of disease and the variance explained. We can also compute many indices of predictive power, such as AUC, discrimination slope, reclassification improvement and the proportion of cases explained by specific percentile of population at the highest risk. Hence the concept of variance explained provides a unifying framework that connects different approaches to prediction model assessment. Although the methods presented are primarily motivated and demonstrated using genetic markers, they can also be applied to other biomarkers or environmental risk factors.

The methodology described here can be applied to case-control studies, as long as the overall probability of outcome and variance explained are known. By supplying the overall disease probability, the appropriate absolute risks and reclassification indices can be computed. This is verified by our simulation which is equivalent to a cohort study. Using the variance explained derived from allele frequencies and ORs, all the prediction metrics and plots from our theoretical calculations approximates the simulation results very well. Another advantage of risk prediction based on the variance explained framework is that it can be extended to deal with markers in LD, haplotypes or multilocus genotypes. These extensions will be discussed elsewhere (So et al., submitted [10]).

### A note on the optimality of the ROC curve

Recently Lu and Elston [4] have suggested a way to construct the optimal ROC curve, based on likelihood ratios. An optimal ROC curve is one that maximizes the true positive rate for any fixed value of false positive rate and hence gives the maximum AUC. Another method to construct the optimal ROC curve is simply to use the risk score function or the predicted probability of disease, Pr(disease| test result). This method is perhaps less recognized and has not been mentioned in Lu and Elston [4]. It has been shown that the risk score is a monotone increasing function of likelihood ratios, and therefore also gives the optimal ROC curve [23]. In our methodology, construction of the ROC curves (and AUC) are all based on the actual predicted risks, or equivalently the liability score, which is a monotone increasing function of the actual risks. As a result, the ROC curve produced by our method should be optimal by definition. In practice, the “optimality” will depend on whether the risks are specified correctly by the most appropriate model. Our approach is to assume an underlying normal liability distribution. Another common approach is to assume a logistic model, which is the same as assuming ORs are multiplicative. These 2 models (normal Vs logistic) are however very close, and in reality it is difficult to tell which one is closer to the truth.

### On measures of predictive power and the limitations of AUC

Broadly speaking, individualized estimates of disease risk serves two main purposes. One is accurate classification of individuals into two distinct groups, diseased or not diseased. Such high levels of accuracy in classification are required, for example in disease diagnosis or identifying subgroups of people for costly and/or invasive screening procedures or interventions. The actual disease risks are relatively unimportant. In this case AUC is an appropriate measure of predictive ability.

Another purpose is to better stratify people into risk categories and offer different management or screening strategies according to the level of predicted risks. The intervention or screening plans are typically less expensive or risky. For example, subjects having a 10-year risk of breast cancer over a certain percentage may be recommended for mammography screening. In that case, the increase in AUC by adding genetic markers to the prediction model may not be the primary concern. Instead, it is more pertinent to know how well an individual may be reclassified into the “screening” and “no screening” groups. This idea of using the reclassification concept to assess a predictive model has been discussed in previous studies [6], [9].

AUC has been widely used to measure the ability of risk prediction for sets of genetic variants and other biomarkers. As mentioned previously, there are limitations in just using AUC to quantify the predictive power of a test. More detailed discussions on the shortcomings are presented elsewhere [6], [7], [24]. In brief, AUC is not a function of the actual predicted risks. As an extreme example described by Cook (2007), a risk model that assigns a risk of 0.52 to all cases and 0.51 to all non-cases achieved perfect discrimination but is not clinically useful. The benefit of new markers in reclassification cannot be captured by AUC as it contains no information about the predicted risks. AUC only depends on the rank of the test results (or the model risks) and ignore the magnitude of differences. If all the predicted risks are multiplied by a factor of 10, the ranks and hence the ROC curve or AUC will stay unchanged, but the clinical impact is very different [24].

Another criticism concerns the interpretation of AUC. AUC equals the probability that the risk of a case is higher than a non-case. This is however not directly clinically relevant since physicians do not see patients in pairs and it is not necessary to decide which person in a pair will develop the disease.

Moreover, the ROC curve and AUC considers all possible cutoffs for the test results or model risks and the specificity and sensitivity at each cutoff. As a result, no pre-specified cutoff is required. However, if there exist established risk thresholds, then a large part of the ROC curve will in fact be derived from cutoff points not of clinical interest.

We reckon that a more comprehensive assessment of prediction model performance may be achieved by complementing AUC with other available prediction indices and graphs, such as NRI, IDI and risk distribution plots, particularly when there exist meaningful risk thresholds. If there are no such thresholds, NRI may not be useful, but AUC, IDI and distribution of risks in the population can still be evaluated. The practical choice or emphasis on which metric is dependent on the clinical context.

### Areas not addressed

Several areas have not been addressed in this study. We have not considered model calibration, which is a measure of how close the predicted probabilities of outcome match the actual probabilities. Calibration may be assessed by dividing subjects into deciles of risk and compare the mean predicted risk with the actual proportion of outcomes in each decile. The goodness of fit may be tested by a chi-square test, also known as the Hosmer-Lemeshow test [25]. The predictiveness curve can also be used to visualize the goodness-of-fit [7]. We have assumed perfect calibration when deriving the results. Interaction between SNPs, for example, may affect calibration. Exploration of interactions will be a useful next step after the single-SNP analyses in GWAS.

The measured liability is assumed to be normally distributed. If we consider a number of SNPs with small effect sizes (as is the case in many complex diseases), the normal assumption is usually acceptable. However, if a disease has one or a few variants with particularly large effects, the normal assumption may not hold. For example, consider a disease with a biallelic locus showing large effects and ten other SNPs with modest effects. The resulting measured liability can be regarded as a mixture of three normal distributions. The mixing proportion will depends on the genotype frequencies of the large-effect locus. The analytic computation of predictive indices will be much more complex. Simulations may need to be performed to assess the predictive power of genetic variants in complicated cases. As an example, for Alzheimer's disease the APOE locus exerts a large effect compared to other loci [26], hence the normal assumption is more dubious and analytic calculations of predictive metrics may not be very accurate.

Another potential problem is that the effect size measures may not be accurate. For example, the effect size of the top significant results in a GWAS may be subject to the “winner's curse”, leading to overestimation of OR. Methods to correct for this bias were described elsewhere [27], [28], [29]. In addition, sometimes cases and controls may not be representative of the general population. For example, some studies may recruit cases enriched for family history or more extreme phenotypes. For the applications in the current study, the effect sizes are based on combining the initial GWAS and replication samples, and the bias should in general be small. As for the practical value of a single GWAS in genetic risk prediction, Wray et al. [30] used simulated case-control datasets to consider the predictive performance of selected markers.

As described before, an important aim of risk prediction models is to stratify people into appropriate risk categories so they may be subject to different interventions or screening strategies. We have not discussed issues regarding the determination of risk thresholds. We need to evaluate the associated costs and benefits before making such a choice. More mathematical treatment based on a decision theory framework may be found for example in [31]. In addition, the costs and benefits may vary with age or other environmental factors and may vary in different populations. In practice, medical decision making will need to involve the patient's own values and tolerance for risks as well. A recent study by Gail [32] provided an example on how different metrics based on public health considerations may be used to assess the performance of a breast cancer prediction model with genetic markers.

We have used NRI to assess how a new test will improve the re-classification of subjects into risk categories. The NRI may also be viewed as the difference in expected loss between the new test and the old. Recalling the formula for NRI,
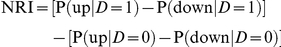
in fact all the four components are given equal weighting. For example, the loss incurred by putting a diseased individual in a lower risk category is assumed to be identical to the loss incurred by putting a healthy person in a higher risk category. In practice, this may not be the most appropriate. For instance, we may consider that putting an affected person into a lower risk category and hence *not* giving him/her treatment [the component P(down|*D* = 1)] is *worse* than erroneously moving up the risk category of a healthy individual [the component P(up|*D* = 0)] and treating the person. This may be true if the morbidity or mortality of the disease is much greater than the side-effects from treatment. To tackle this problem one can for example incorporate different loss functions for the components. This may be done within our framework by weighing the four components differently. In a similar vein, Gail [32] considered different loss functions for TP, TN, FP and FN and evaluated the expected loss for mammography screening if the risk threshold was set at a certain optimal level. As described previously, we can derive all four components in a 2×2 classification table (Table 1) and the expected loss can also be obtained readily by assigning appropriate losses to each component.

Another noteworthy limitation is that if there exists more than one risk bin, NRI does not consider the number of risk categories changed upon adding the new marker. For example, say there are three risk categories, placing a case in the lowest risk bin and the second-lowest bin will have the same effect on NRI. To correct this problem, we may consider giving numeric scores that reflect the number of categories shifted, as suggested in [28]. For example, moving up a person who develops the event from category 1 (lowest risk) to category 3 (highest risk) will receive a higher score than just moving up to category 2 (intermediate risk). Again, our framework can be readily extended to deal with this problem, as we can calculate the probability of changing from any one risk category to another using bivariate integration.

We have mainly considered lifetime risk when calculating predictive indices. In clinical practice the risk in a given period of time may be more relevant than the lifetime risk. For example, the ATP III guideline quoted above relies on 10-year risk of coronary heart disease. The use of tamoxifen for breast cancer prevention is dependent on the 5-year risk of disease [33], [34]. It is also worth noting that the absolute risk of disease is reduced by the chance that an individual dies of other causes prior to developing the disease. Our statistical framework can be extended to deal with risks in a specified period of time (for individuals of a certain age). In that case, the inputs should be carefully specified. The disease probability and the relative risks *in the specified period of time* should be provided to compute the correct variance explained. The rest of the calculations remain unchanged. Concepts of absolute risk estimation were discussed for example in [35]. We describe in a separate paper [36] the detailed methodologies to derive age-conditional risk estimates given a follow-up period, accounting for competing risks in the context of genetic association studies.

We have not considered the variability of the predictive metrics in this study. It should be noted that the disease probability and the variance explained by known variants are both subject to variations, and so will be the predictive metrics derived from these two quantities. One should be careful in interpretation of the results if the disease probability or effect sizes of variants are estimated from small sample sizes.

We noticed a recent study on AUC estimation in the context of genetic risk prediction [37]. Their study is also based on a liability threshold model, and they have derived an analytic formula to approximate the maximum AUC achieved when all genetic loci are found (at heritability or at fractions of it). Their formula described is similar to ours under the section on approximation of AUC by the binormal ROC curve. However, apart from this, other methodologies presented here have not been described before. Besides the approximation formula based on normal distributions in cases and controls, we also provide the exact formula for AUC as well as formulas to derive all 4 cells in the 2×2 classification table (given any threshold). Hence we are able to draw the ROC curve based on analytic methods. Also, not only are we able to derive the AUC when all heritability has been explained, but we may also calculate the AUC directly given the allele frequencies and effect sizes of a set of known susceptibility loci. We have also argued for other measures and graphs to evaluate the predictive performance of models and provide analytic formulas to obtain all relevant indices or plots.

Evaluation of a risk prediction model is no simple task, and inevitably we cannot perfectly deal with every complexity involved. Nevertheless, we hope the current study will stimulate more thoughts on the proper assessment of prediction models and provide a convenient and useful methodology for researchers to assess the predictive ability of sets of susceptibility loci. Programs (written in R) to implement the methodology presented in this paper are available at https://sites.google.com/site/honcheongso/software/pred-metrics.

## Supporting Information

Figure S1 Graphs showing risk distribution and predictive power of known susceptibility variants for bipolar disorder.
(0.47 MB TIF)

Figure S2 Graphs showing risk distribution and predictive power of known susceptibility variants for prostate cancer.
(0.24 MB TIF)

Figure S3 Graphs showing risk distribution and predictive power of known susceptibility variants for coronary artery disease.
(0.48 MB TIF)

Figure S4 Graphs showing risk distribution and predictive power of known susceptibility variants for Crohn's disease.
(0.47 MB TIF)

Figure S5 Graphs showing risk distribution and predictive power of known susceptibility variants for type 1 diabetes mellitus.
(0.47 MB TIF)

Figure S6 Graphs showing risk distribution and predictive power of known susceptibility variants for type 2 diabetes mellitus.
(0.47 MB TIF)

Figure S7 Graphs showing risk distribution and predictive power of known susceptibility variants for schizophrenia.
(0.47 MB TIF)

Figure S8 Graphs showing risk distribution and predictive power of known susceptibility variants for systemic lupus erythematosus (SLE).
(0.48 MB TIF)

Table S1 List of the risk allele frequencies (RAF) and odds ratios (OR) of 30 established susceptibility variants for prostate cancer.
(0.05 MB DOC)

Table S2 Predictive indices from simulations (sim) compared to theoretical estimates (theo).
(0.07 MB DOC)

Table S3 NRI and its components in simulations and theoretical estimates.
(0.05 MB DOC)

Text S1 Supplementary methods.
(0.11 MB PDF)

## References

[1] Jakobsdottir J, Gorin MB, Conley YP, Ferrell RE, Weeks DE (2009). Interpretation of genetic association studies: markers with replicated highly significant odds ratios may be poor classifiers.. PLoS Genet.

[2] Kraft P, Wacholder S, Cornelis MC, Hu FB, Hayes RB (2009). Beyond odds ratios–communicating disease risk based on genetic profiles.. Nat Rev Genet.

[3] Janssens AC, Aulchenko YS, Elefante S, Borsboom GJ, Steyerberg EW (2006). Predictive testing for complex diseases using multiple genes: fact or fiction?. Genet Med.

[4] Lu Q, Elston RC (2008). Using the optimal receiver operating characteristic curve to design a predictive genetic test, exemplified with type 2 diabetes.. Am J Hum Genet.

[5] Janssens AC, van Duijn CM (2008). Genome-based prediction of common diseases: advances and prospects.. Hum Mol Genet.

[6] Cook NR (2007). Use and misuse of the receiver operating characteristic curve in risk prediction.. Circulation.

[7] Pepe MS, Feng Z, Huang Y, Longton G, Prentice R (2008). Integrating the predictiveness of a marker with its performance as a classifier.. Am J Epidemiol.

[8] Pepe MS, Janes H, Longton G, Leisenring W, Newcomb P (2004). Limitations of the odds ratio in gauging the performance of a diagnostic, prognostic, or screening marker.. Am J Epidemiol.

[9] Pencina MJ, D'Agostino RB, D'Agostino RB, Vasan RS (2008). Evaluating the added predictive ability of a new marker: from area under the ROC curve to reclassification and beyond.. Stat Med.

[10] So HC, Gui AH, Cherny SS, Sham PC (2010). Evaluating the heritability explained by known susceptibility variants: a survey of ten complex diseases..

[11] Aitken AC

[12] Pearson K (1903). Mathematical contributions to the theory of evolution. XI..

[13] Pepe MS (2003). The statistical evaluation of medical tests for classification and prediction.

[14] Pharoah PD, Antoniou A, Bobrow M, Zimmern RL, Easton DF (2002). Polygenic susceptibility to breast cancer and implications for prevention.. Nat Genet.

[15] Clayton DG (2009). Prediction and interaction in complex disease genetics: experience in type 1 diabetes.. PLoS Genet.

[16] Wacholder S, Hartge P, Prentice R, Garcia-Closas M, Feigelson HS (2010). Performance of common genetic variants in breast-cancer risk models.. N Engl J Med.

[17] Lorenz M (1905). Methods of measuring the concentration of wealth..

[18] Yates JF (1982). External correspondence: Decompositions of the mean probability score.. Organizational Behavior and Human Performance.

[19] Pepe MS, Feng Z, Gu JW (2008). Comments on ‘Evaluating the added predictive ability of a new marker: From area under the ROC curve to reclassification and beyond’ by M. J. Pencina et al., Statistics in Medicine (DOI: 10.1002/sim.2929).. Stat Med.

[20] Zhang J, Yu KF (1998). What's the relative risk? A method of correcting the odds ratio in cohort studies of common outcomes.. JAMA.

[21] Aldrich JH, Nelson FD (1984). Linear probability, logit, and probit models.

[22] Janssens AC, Moonesinghe R, Yang Q, Steyerberg EW, van Duijn CM (2007). The impact of genotype frequencies on the clinical validity of genomic profiling for predicting common chronic diseases.. Genet Med.

[23] McIntosh MW, Pepe MS (2002). Combining several screening tests: optimality of the risk score.. Biometrics.

[24] Pepe MS, Janes HE (2008). Gauging the performance of SNPs, biomarkers, and clinical factors for predicting risk of breast cancer.. J Natl Cancer Inst.

[25] Lemeshow S, Hosmer DW (1982). A review of goodness of fit statistics for use in the development of logistic regression models.. Am J Epidemiol.

[26] Farrer L, Cupples L, Haines J, Hyman B, Kukull W (1997). Effects of age, sex, and ethnicity on the association between apolipoprotein E genotype and Alzheimer disease. A meta-analysis. APOE and Alzheimer Disease Meta Analysis Consortium.. JAMA.

[27] Garner C (2007). Upward bias in odds ratio estimates from genome-wide association studies.. Genet Epidemiol.

[28] Ghosh A, Zou F, Wright FA (2008). Estimating odds ratios in genome scans: an approximate conditional likelihood approach.. Am J Hum Genet.

[29] Zhong H, Prentice RL (2008). Bias-reduced estimators and confidence intervals for odds ratios in genome-wide association studies.. Biostatistics.

[30] Wray NR, Goddard ME, Visscher PM (2007). Prediction of individual genetic risk to disease from genome-wide association studies.. Genome Res.

[31] Gail MH, Pfeiffer RM (2005). On criteria for evaluating models of absolute risk.. Biostatistics.

[32] Gail MH (2009). Value of adding single-nucleotide polymorphism genotypes to a breast cancer risk model.. J Natl Cancer Inst.

[33] Fisher B, Costantino JP, Wickerham DL, Redmond CK, Kavanah M (1998). Tamoxifen for prevention of breast cancer: report of the National Surgical Adjuvant Breast and Bowel Project P-1 Study.. J Natl Cancer Inst.

[34] Fisher B, Costantino JP, Wickerham DL, Cecchini RS, Cronin WM (2005). Tamoxifen for the prevention of breast cancer: current status of the National Surgical Adjuvant Breast and Bowel Project P-1 study.. J Natl Cancer Inst.

[35] Gail MH, Rebbeck TR, Ambrosone CB, Shields PG (2008). Models of absolute risk: interpretation, estimation, validation and application.. Molecular epidemiology: applications in cancer and other human diseases.

[36] So HC, Sham PC (2010). Effect Size Measures in Genetic Association Studies and Age-Conditional Risk Prediction.. Hum Hered.

[37] Wray NR, Yang J, Goddard ME, Visscher PM (2010). The genetic interpretation of area under the ROC curve in genomic profiling.. PLoS Genet.

