# Supplementary figures and images for: A Unifying Framework for Evaluating the Predictive Power of Genetic Variants Based on the Level of Heritability Explained
Source: PLoS Genet. 2010 Dec 2;6(12):e1001230. doi: 10.1371/journal.pgen.1001230 (PMC2996330; doi:10.1371/journal.pgen.1001230)

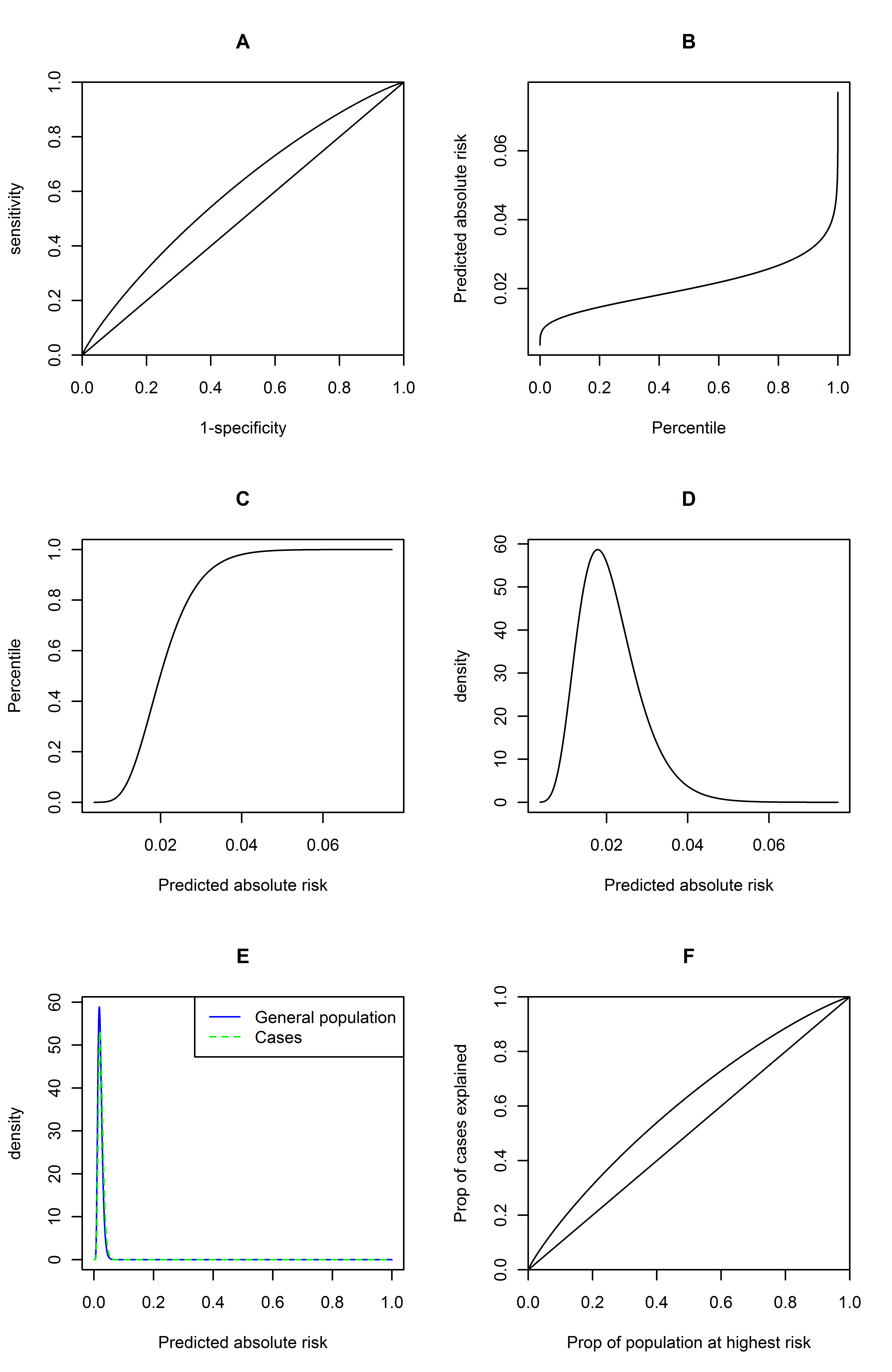

Supplement: Figure S1 — Graphs showing risk distribution and predictive power of known susceptibility variants for bipolar disorder. (0.47 MB TIF) [file pgen.1001230.s001.tif]

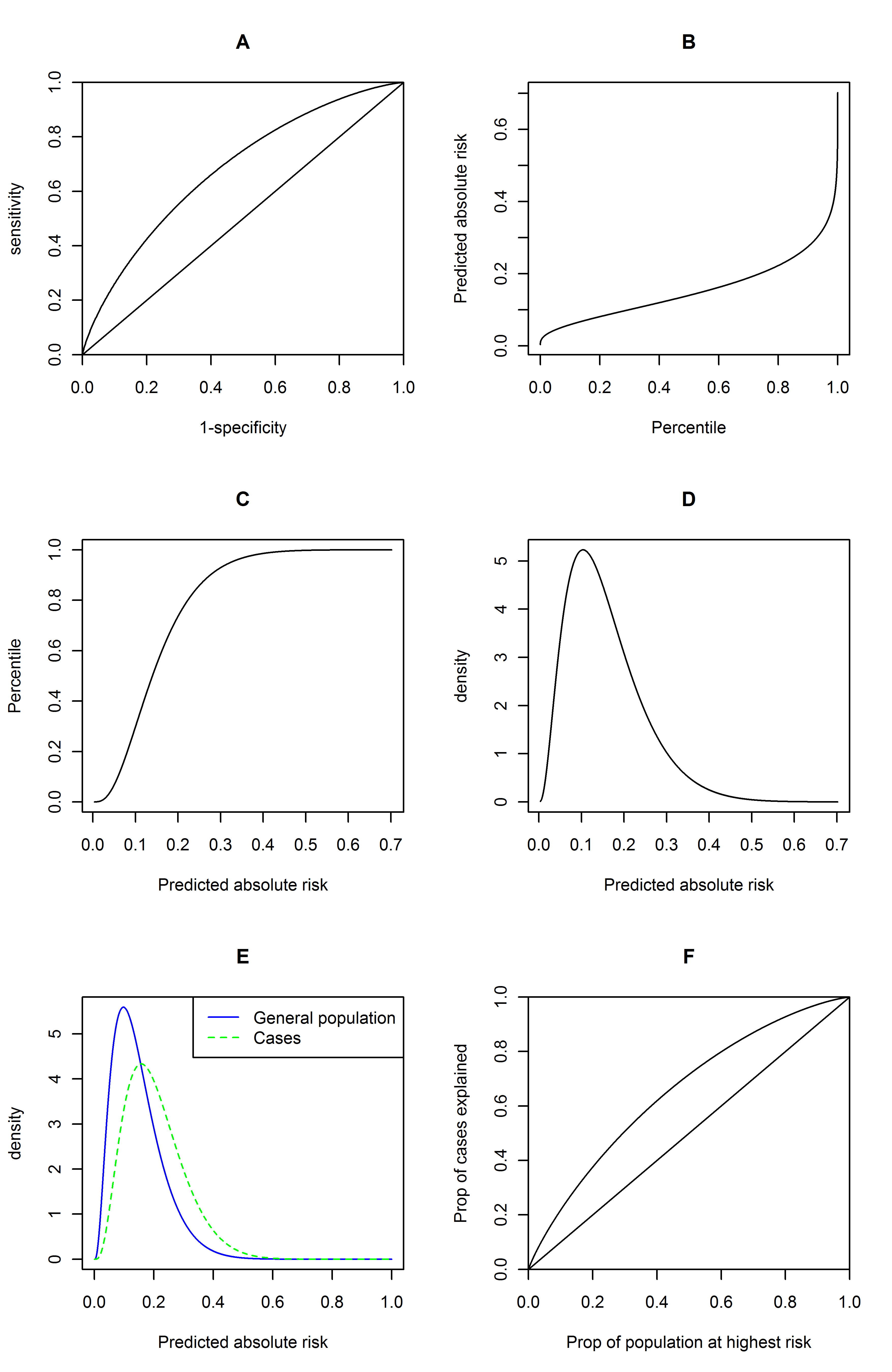

Supplement: Figure S2 — Graphs showing risk distribution and predictive power of known susceptibility variants for prostate cancer. (0.24 MB TIF) [file pgen.1001230.s002.tif]

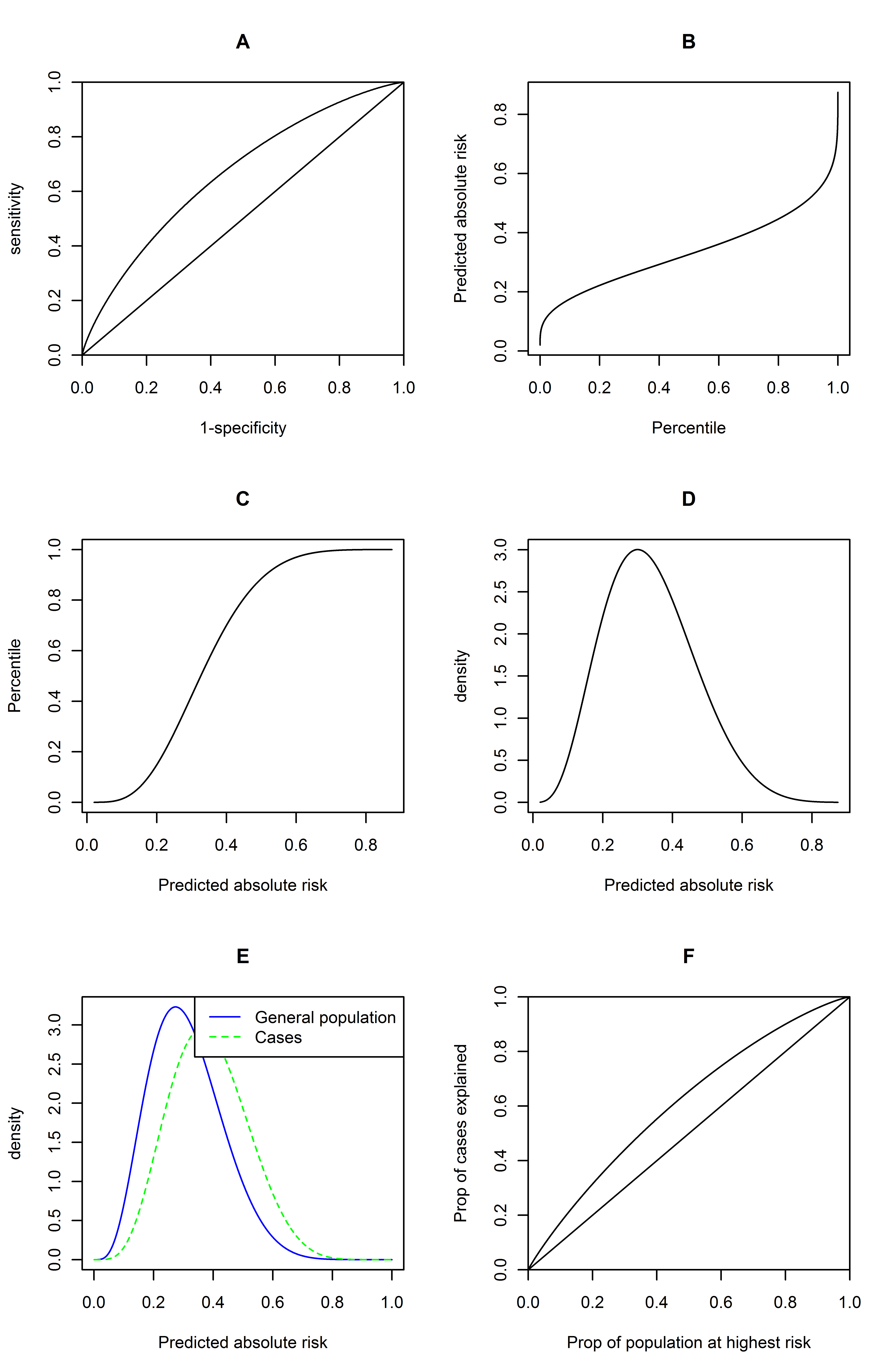

Supplement: Figure S3 — Graphs showing risk distribution and predictive power of known susceptibility variants for coronary artery disease. (0.48 MB TIF) [file pgen.1001230.s003.tif]

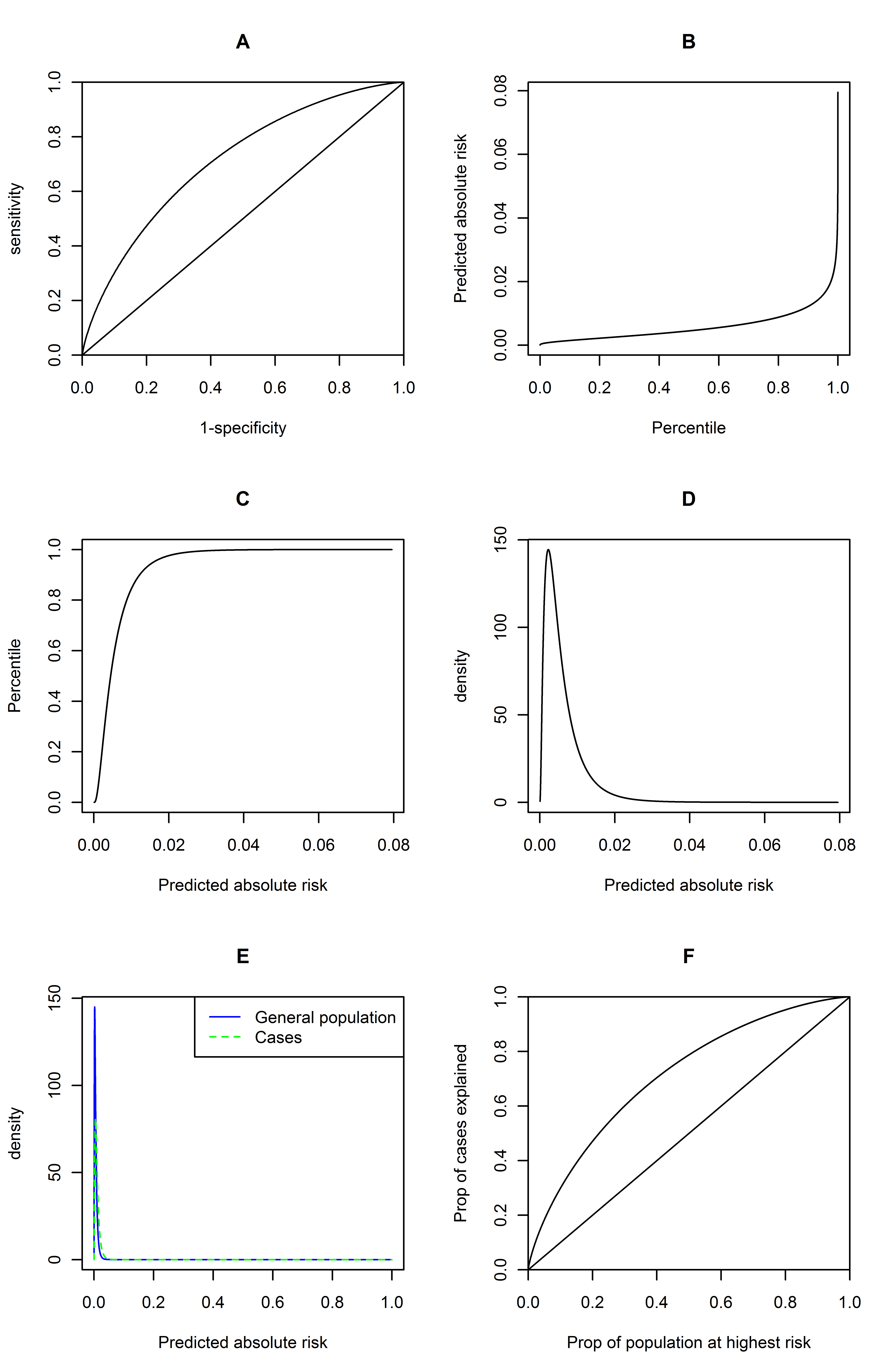

Supplement: Figure S4 — Graphs showing risk distribution and predictive power of known susceptibility variants for Crohn's disease. (0.47 MB TIF) [file pgen.1001230.s004.tif]

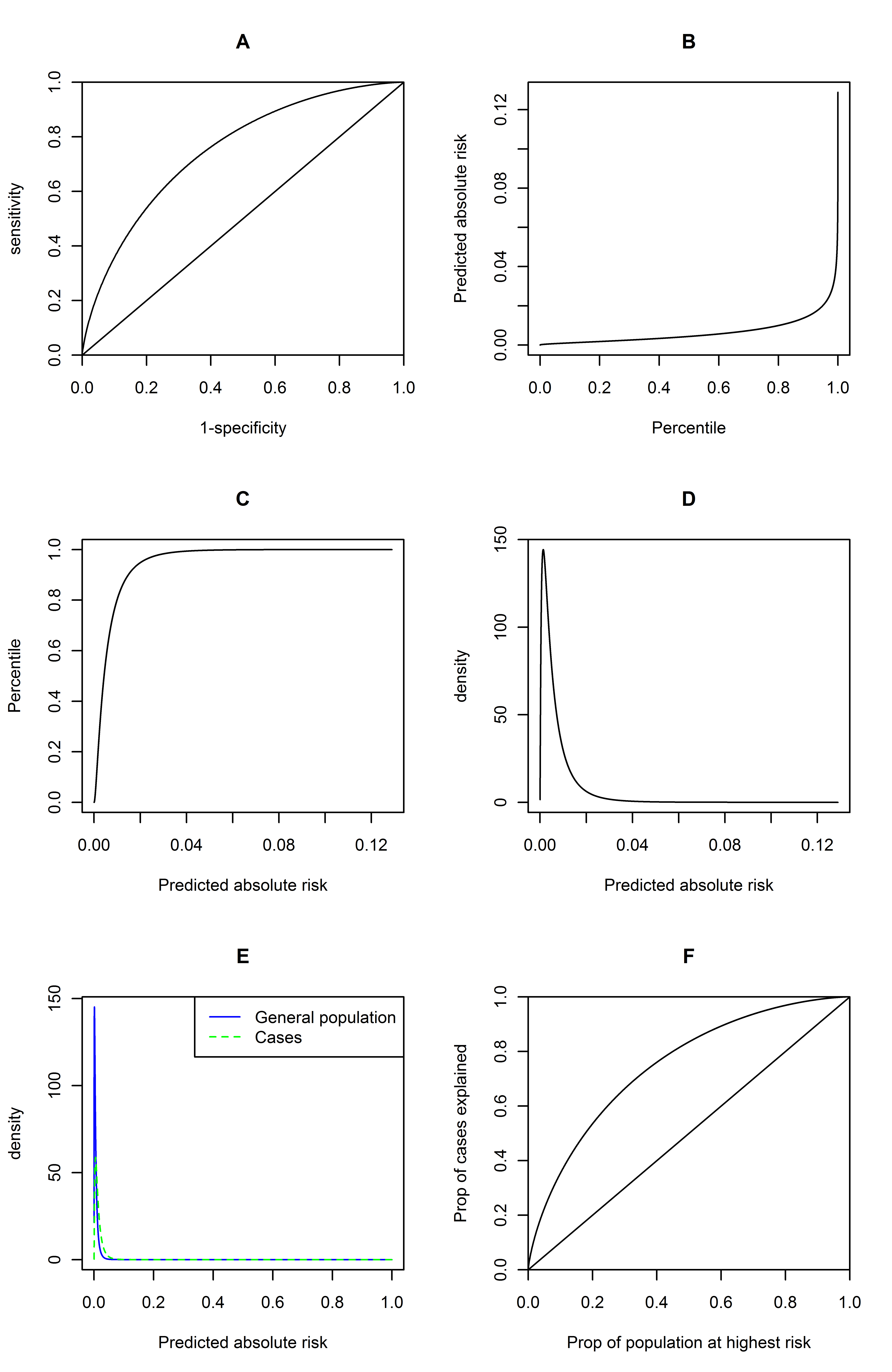

Supplement: Figure S5 — Graphs showing risk distribution and predictive power of known susceptibility variants for type 1 diabetes mellitus. (0.47 MB TIF) [file pgen.1001230.s005.tif]

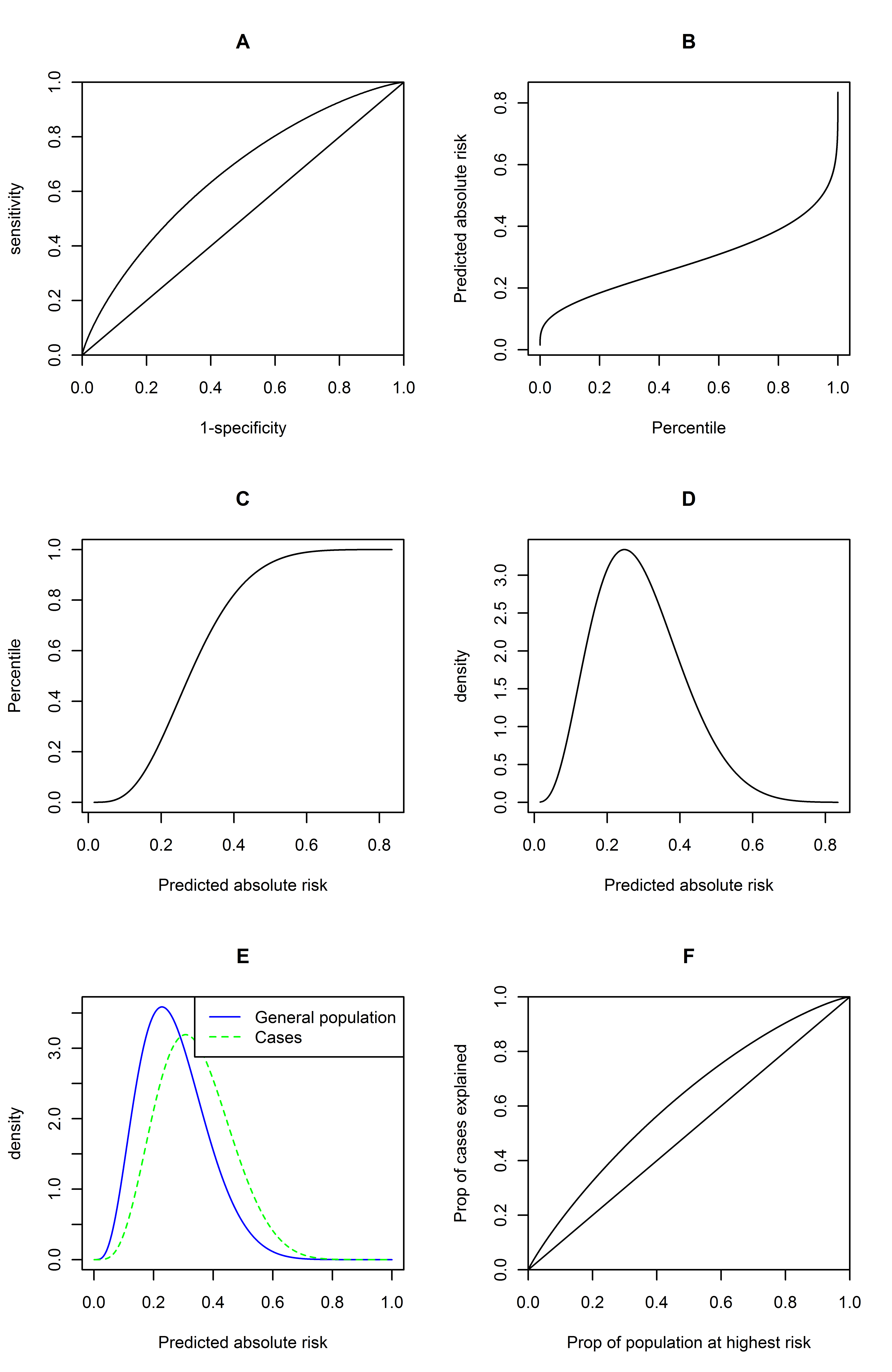

Supplement: Figure S6 — Graphs showing risk distribution and predictive power of known susceptibility variants for type 2 diabetes mellitus. (0.47 MB TIF) [file pgen.1001230.s006.tif]

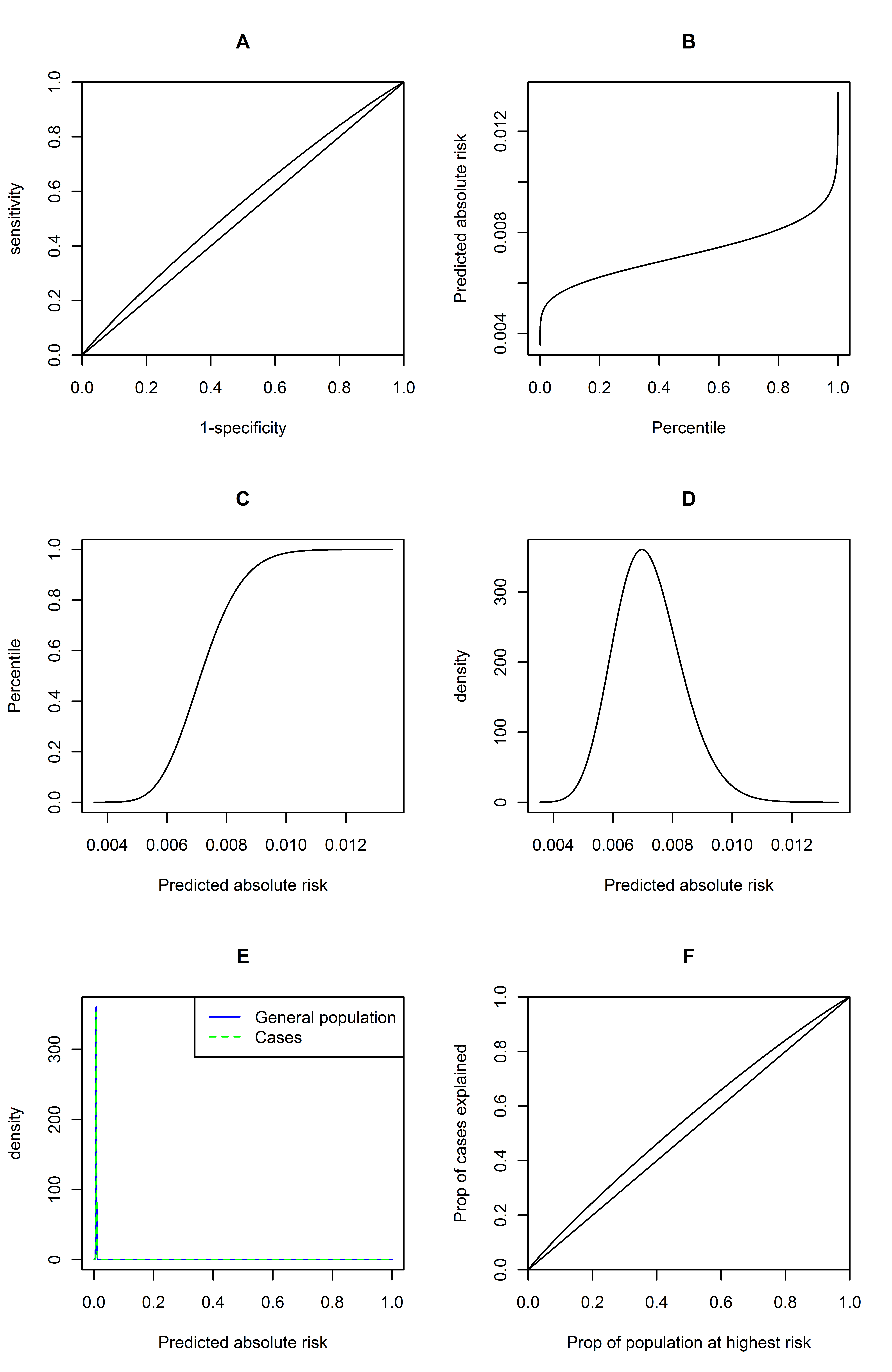

Supplement: Figure S7 — Graphs showing risk distribution and predictive power of known susceptibility variants for schizophrenia. (0.47 MB TIF) [file pgen.1001230.s007.tif]

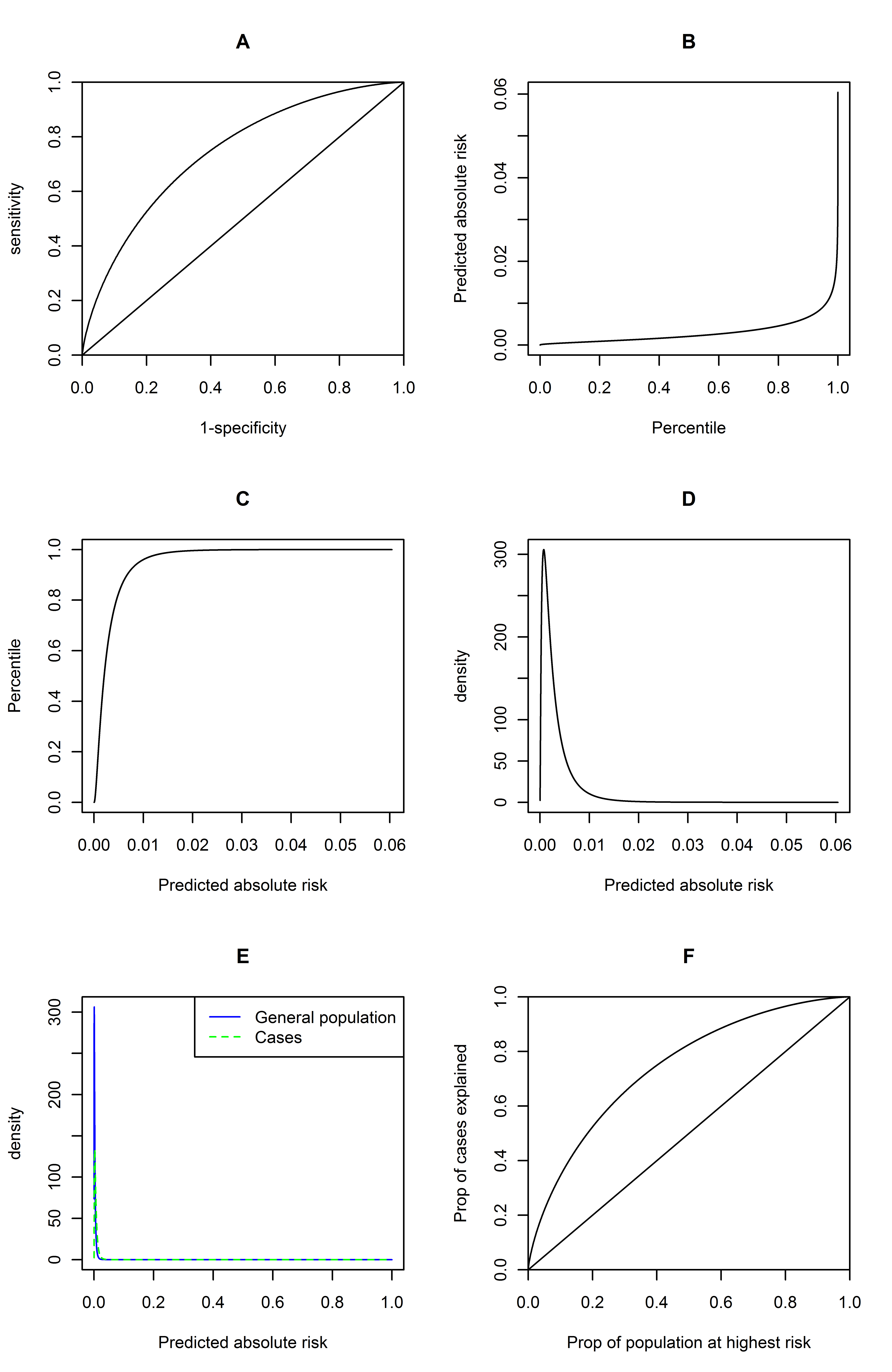

Supplement: Figure S8 — Graphs showing risk distribution and predictive power of known susceptibility variants for systemic lupus erythematosus (SLE). (0.48 MB TIF) [file pgen.1001230.s008.tif]
