# Supplementary material for: A Unifying Framework for Evaluating the Predictive Power of Genetic Variants Based on the Level of Heritability Explained
Source: PLoS Genet. 2010 Dec 2;6(12):e1001230. doi: 10.1371/journal.pgen.1001230 (PMC2996330; doi:10.1371/journal.pgen.1001230)
Supplement: Table S1 — List of the risk allele frequencies (RAF) and odds ratios (OR) of 30 established susceptibility variants for prostate cancer. (0.05 MB DOC) [file pgen.1001230.s009.doc]

Table S1 List of the risk allele frequencies (RAF) and odds ratios (OR) of 30 established susceptibility variants for prostate cancer

| Number | SNPs | RAF | OR |
| --- | --- | --- | --- |
| 1 | rs10993994 | 0.4 | 1.25 |
| 2 | rs7127900 | 0.2 | 1.22 |
| 3 | rs7931342 | 0.51 | 1.19 |
| 4 | rs11228565 | 0.2 | 1.23 |
| 5 | rs4430796 | 0.49 | 1.22 |
| 6 | rs7501939 | 0.57 | 1.41 |
| 7 | rs1859962 | 0.46 | 1.20 |
| 8 | rs8102476 | 0.54 | 1.12 |
| 9 | rs2735839 | 0.85 | 1.20 |
| 10 | rs5759167 | 0.53 | 1.16 |
| 11 | rs721048 | 0.19 | 1.15 |
| 12 | rs1465618 | 0.23 | 1.08 |
| 13 | rs12621278 | 0.94 | 1.33 |
| 14 | rs2660753 | 0.11 | 1.18 |
| 15 | rs10934853 | 0.28 | 1.12 |
| 16 | rs12500426 | 0.46 | 1.08 |
| 17 | rs17021918 | 0.65 | 1.11 |
| 18 | rs7679673 | 0.55 | 1.10 |
| 19 | rs9364554 | 0.29 | 1.17 |
| 20 | rs6465657 | 0.46 | 1.12 |
| 21 | rs5945572 | 0.35 | 1.23 |
| 22 | rs1512268 | 0.45 | 1.18 |
| 23 | rs12543663 | 0.31 | 1.08 |
| 24 | rs10086908 | 0.7 | 1.15 |
| 25 | rs1016343 | 0.2 | 1.21 |
| 26 | rs13252298 | 0.7 | 1.19 |
| 27 | rs6983561 | 0.03 | 1.47 |
| 28 | rs620861 | 0.63 | 1.11 |
| 29 | rs6983267 | 0.51 | 1.26 |
| 30 | rs10090154 | 0.09 | 1.47 |
