# Supplementary material for: A Unifying Framework for Evaluating the Predictive Power of Genetic Variants Based on the Level of Heritability Explained
Source: PLoS Genet. 2010 Dec 2;6(12):e1001230. doi: 10.1371/journal.pgen.1001230 (PMC2996330; doi:10.1371/journal.pgen.1001230)
Supplement: Table S2 — Predictive indices from simulations (sim) compared to theoretical estimates (theo). (0.07 MB DOC) [file pgen.1001230.s010.doc]

Table S2 Predictive indices from simulations (sim) compared to theoretical estimates (theo)

|  | No loci | K | Vg | AUC accurate | AUC approx | AUC approx2 | Prop cases exp 0.1 | Prop cases exp 0.2 | Prop cases exp 0.5 | Var  of risk | Var of risk to max | Mean risk in cases | Mean risk in noncases | Mean Risk Diff |
| --- | --- | --- | --- | --- | --- | --- | --- | --- | --- | --- | --- | --- | --- | --- |
| Theo | 10 | 0.0049 | 0.0217 | 0.619 | 0.619 | 0.620 | 0.194 | 0.338 | 0.667 | 4.72E-06 | 0.0010 | 0.0059 | 0.0049 | 0.0010 |
| Sim |  |  |  | 0.620 |  |  | 0.192 | 0.340 | 0.666 | 4.58E-06 | 0.0009 | 0.0058 | 0.0048 | 0.0009 |
| Theo | 10 | 0.0096 | 0.0252 | 0.619 | 0.619 | 0.620 | 0.193 | 0.337 | 0.666 | 1.79E-05 | 0.0019 | 0.0115 | 0.0096 | 0.0019 |
| Sim |  |  |  | 0.619 |  |  | 0.191 | 0.336 | 0.668 | 1.82E-05 | 0.0019 | 0.0115 | 0.0097 | 0.0019 |
| Theo | 10 | 0.1047 | 0.0610 | 0.619 | 0.619 | 0.621 | 0.180 | 0.320 | 0.652 | 1.67E-03 | 0.0179 | 0.1207 | 0.1028 | 0.0179 |
| Sim |  |  |  | 0.618 |  |  | 0.180 | 0.319 | 0.648 | 1.64E-03 | 0.0175 | 0.1203 | 0.1028 | 0.0175 |
| Theo | 20 | 0.0050 | 0.0286 | 0.636 | 0.636 | 0.637 | 0.211 | 0.360 | 0.690 | 6.65E-06 | 0.0013 | 0.0064 | 0.0050 | 0.0013 |
| Sim |  |  |  | 0.630 |  |  | 0.204 | 0.350 | 0.678 | 6.89E-06 | 0.0014 | 0.0064 | 0.0051 | 0.0013 |
| Theo | 20 | 0.0104 | 0.0336 | 0.636 | 0.636 | 0.637 | 0.209 | 0.359 | 0.689 | 2.81E-05 | 0.0027 | 0.0131 | 0.0104 | 0.0027 |
| Sim |  |  |  | 0.636 |  |  | 0.210 | 0.363 | 0.684 | 2.72E-05 | 0.0026 | 0.0128 | 0.0102 | 0.0027 |
| Theo | 20 | 0.0999 | 0.0631 | 0.637 | 0.636 | 0.638 | 0.194 | 0.340 | 0.675 | 2.04E-03 | 0.0227 | 0.1204 | 0.0976 | 0.0227 |
| Sim |  |  |  | 0.634 |  |  | 0.193 | 0.339 | 0.669 | 1.99E-03 | 0.0221 | 0.1196 | 0.0976 | 0.0220 |
| Theo | 30 | 0.0049 | 0.0442 | 0.668 | 0.667 | 0.669 | 0.246 | 0.406 | 0.733 | 1.03E-05 | 0.0021 | 0.0070 | 0.0049 | 0.0021 |
| Sim |  |  |  | 0.664 |  |  | 0.247 | 0.405 | 0.726 | 1.03E-05 | 0.0021 | 0.0070 | 0.0049 | 0.0021 |
| Theo | 30 | 0.0109 | 0.0527 | 0.668 | 0.667 | 0.669 | 0.244 | 0.404 | 0.732 | 4.98E-05 | 0.0046 | 0.0155 | 0.0108 | 0.0046 |
| Sim |  |  |  | 0.667 |  |  | 0.244 | 0.404 | 0.725 | 4.94E-05 | 0.0046 | 0.0154 | 0.0108 | 0.0046 |
| Theo | 30 | 0.0960 | 0.0971 | 0.670 | 0.667 | 0.672 | 0.224 | 0.381 | 0.718 | 3.05E-03 | 0.0352 | 0.1278 | 0.0927 | 0.0352 |
| Sim |  |  |  | 0.664 |  |  | 0.221 | 0.376 | 0.706 | 2.86E-03 | 0.0329 | 0.1256 | 0.0927 | 0.0329 |

K : overall disease prevalence ; Vg , variance explained
