# Supplementary material for: A Unifying Framework for Evaluating the Predictive Power of Genetic Variants Based on the Level of Heritability Explained
Source: PLoS Genet. 2010 Dec 2;6(12):e1001230. doi: 10.1371/journal.pgen.1001230 (PMC2996330; doi:10.1371/journal.pgen.1001230)
Supplement: Table S3 — NRI and its components in simulations and theoretical estimates. (0.05 MB DOC) [file pgen.1001230.s011.doc]

Table S3 NRI and its components in simulations and theoretical estimates

|  | Actual K | Vold | Vnew | Risk threshold | Pr.up.case | Pr.down.case | Pr.up.unaff | Pr.down.unaff | NRI |
| --- | --- | --- | --- | --- | --- | --- | --- | --- | --- |
| Sim | 0.0050 | 0.0285 | 0.0443 | 0.0075 | 0.125 | 0.063 | 0.068 | 0.049 | 0.043 |
| Theo |  |  |  | 0.0075 | 0.141 | 0.055 | 0.075 | 0.047 | 0.058 |
| Sim |  |  |  | 0.01 | 0.097 | 0.029 | 0.040 | 0.019 | 0.046 |
| Theo |  |  |  | 0.01 | 0.111 | 0.026 | 0.045 | 0.016 | 0.057 |
| Sim | 0.0109 | 0.0298 | 0.0528 | 0.015 | 0.157 | 0.066 | 0.090 | 0.063 | 0.065 |
| Theo |  |  |  | 0.015 | 0.167 | 0.067 | 0.095 | 0.064 | 0.069 |
| Sim |  |  |  | 0.02 | 0.135 | 0.031 | 0.059 | 0.023 | 0.068 |
| Theo |  |  |  | 0.02 | 0.146 | 0.032 | 0.063 | 0.022 | 0.073 |
| Sim | 0.0958 | 0.0623 | 0.0970 | 0.15 | 0.126 | 0.044 | 0.062 | 0.034 | 0.054 |
| Theo |  |  |  | 0.15 | 0.140 | 0.046 | 0.068 | 0.035 | 0.061 |
| Sim |  |  |  | 0.2 | 0.079 | 0.015 | 0.028 | 0.008 | 0.045 |
| Theo |  |  |  | 0.2 | 0.088 | 0.015 | 0.031 | 0.008 | 0.050 |

Vold: variance explained in the old model ; Vnew, variance explained in the new model;

Pr.up.case: probability of moving *up* risk categories among *cases* ; Pr.down.case, probability of moving *down* risk categories among *cases;* Pr.up.unaff,

probability of moving *up* risk categories among *unaffected* individuals; Pr.down.unaff, probability of moving *down* risk categories among *unaffected* individuals.
