# Supplementary material for: A Unifying Framework for Evaluating the Predictive Power of Genetic Variants Based on the Level of Heritability Explained
Source: PLoS Genet. 2010 Dec 2;6(12):e1001230. doi: 10.1371/journal.pgen.1001230 (PMC2996330; doi:10.1371/journal.pgen.1001230)
Supplement: Text S1 — Supplementary methods. (0.11 MB PDF) [file pgen.1001230.s012.pdf]

Supplementary methods (Text S1) for "Evaluating the predictive power of genetic variants under a variance explained framework"

Hon-Cheong So and Pak C. Sham

1. Approximation of TPR, FPR and AUC by the binormal ROC curve

Denote the measurable liability in affected and unaffected individuals by M_A and $M_{\bar{A}}$ respectively. Suppose

$M_A \sim N(\mu_A, \sigma_A^2)$ and $M_{\bar{A}} \sim N(\mu_{\bar{A}}, \sigma_{\bar{A}}^2)$

The AUC for the binormal ROC curve can be expressed in a simple form [1] :

$$AUC = \Phi\left(\frac{(\mu_A - \mu_{\bar{A}}) / \sigma_A}{\sqrt{1 + (\sigma_A / \sigma_{\bar{A}})^2}} \right)$$

The mean measurable liability in cases, or μ_A , has been derived previously to be aσ^2, where $a = \phi(T) / [1 - \Phi(T)]$ and σ^2 is the variance explained. Since the overall liability is 0,

$$\mu_A K + \mu_{\bar{A}}(1-K) = 0$$

$$a\sigma^2 K + \mu_{\bar{A}}(1-K) = 0$$

$$\mu_{\bar{A}} = \frac{-a\sigma^2 K}{1-K}$$

Hence

$$\mu_A - \mu_{\bar{A}} = a\sigma^2 \left(1 + \frac{K}{1-K}\right)$$

We may assume that the variances in the affected and unaffected groups are approximately equal, especially for more common diseases. AUC can then be approximated by the following formula:

$$AUC = \Phi\left(\frac{a\sigma^2 \left(1 + \frac{K}{1-K}\right) / \sigma_A}{\sqrt{2}} \right)$$

where σ_A is the standard deviation in cases which equals $\sqrt{\sigma^2[1-(1-b)\sigma^2]}$ with $b = 1 - a^2 + aT$. This method of estimating AUC does not involve any numerical integration or simulations and can be easily implemented in a spreadsheet.

Alternatively, to improve the accuracy of AUC estimate, we may calculate $\sigma_A / \sigma_{\bar{A}}$ using the actual standard deviations of liability (derived using the PA formula) in affected and

unaffected groups.

$$\frac{\sigma_A}{\sigma_{\bar{A}}} = \frac{\sqrt{1-(1-b)V}}{\sqrt{1-(1-d)V}}$$

with *b* and *d* as defined before in main text.

2. **Probability density function of predicted risks**

Let *z* denote the quantile of the measurable liability derived from the set of known genes, i.e.

$z = \sigma\Phi^{-1}(p)$ and $R = 1 - \Phi(\frac{T-z}{\sqrt{1-\sigma^2}})$

Then

$$\frac{dR}{dz} = -\phi(\frac{T-z}{\sqrt{1-\sigma^2}})(-\frac{1}{\sqrt{1-\sigma^2}}) = \frac{\phi\left(\frac{T-\sigma\Phi^{-1}(p)}{\sqrt{1-\sigma^2}}\right)}{\sqrt{1-\sigma^2}}$$

$$\frac{dz}{dp} = \frac{\sigma}{\phi(\Phi^{-1}(p))}$$

and $\frac{dR}{dp} = \frac{dR}{dz}\left(\frac{dz}{dp}\right)$

hence *dp/dR* , or the pdf of the absolute risk, can readily be obtained by taking the reciprocal.

Note also the formula to convert R to *p* is

$$p = \Phi\left[\frac{T - \Phi^{-1}(1-R)\sqrt{1-\sigma^2}}{\sigma}\right]$$

Risk distribution in affected and unaffected individuals

Assume that again we wish to predict disease risks given a set of known susceptibility genes. However, in this case we would like to now how the *predicted* risks (not the actual risks, the actual risk can only be 0 or 1 if we know the affection status) will be distributed in affected and unaffected individuals.

R is defined in the same way as above. Again we have

$$\frac{dR}{dz} = -\phi\left(\frac{T-z}{\sqrt{1-\sigma^2}}\right)\left(-\frac{1}{\sqrt{1-\sigma^2}}\right)$$

where σ^2 is the variance explained by the known genes, but the distribution of *z* is different. In affected subjects, *z* may be written as

$$z = \Phi^{-1}(p)\sigma_A + \mu_A$$

where σ_A^2 and μ_A are the variance and mean of the measurable liability for affected individuals. $\mu_A = a\sigma^2$ and $\sigma_A^2 = \sigma^2\,[1\text{-}(1\text{-}b)\,\sigma^2]$ from previous results. *dz/dp* is given by

$$\frac{dz}{dp} = \frac{\sigma_A}{\phi(\Phi^{-1}(p))}$$

dp/dR or the pdf of absolute risk can then be enumerated. For unaffected individuals, the calculation is very similar, only that the mean and variance equals $c\sigma^2$ and $\sigma^2[1\text{-}(1\text{-}d)\sigma^2]$ respectively.

3. Expression for AUC and Area under the curve when proportion of cases explained is plotted against population at the highest risk

We have previously derived the Pr(true positive) for a given percentile cut-off c. Test is defined as positive if the liability score exceeds this cut-off. The sensitivity of the test when the cut-off point is set at c is given by

$$sens(c) = \frac{\Pr(TP \text{ at c})}{K} = \frac{\int_c^1 [1-\Phi(\frac{T-\sigma\Phi^{-1}(p)}{\sqrt{1-\sigma^2}})]dp}{K}$$

Similarly, 1-specificity (or the false positive rate, FPR) is given by

$$1-spec(c) = \frac{\Pr(FP \text{ at c})}{1-K} = \frac{(1-c)-\int_c^1 [1-\Phi(\frac{T-\sigma\Phi^{-1}(p)}{\sqrt{1-\sigma^2}})]dp}{1-K}$$

The AUC is the area under the curve when sensitivity is plotted against 1-specificity. This area is given by

$$AUC = \int_0^1 sens(c)d(1-spec(c))$$

$$\frac{d(1-spec(c))}{dc}$$

$$=\left(\frac{1}{1-K}\right)\frac{d}{dc}\left((1-c)-\int_c^1 [1-\Phi(\frac{T-\sigma\Phi^{-1}(p)}{\sqrt{1-\sigma^2}})]dp\right)$$

$$=\frac{1}{1-K}\left(-1-\frac{d}{dc}\int_c^1 [1-\Phi(\frac{T-\sigma\Phi^{-1}(p)}{\sqrt{1-\sigma^2}})]dp\right)$$

$$=\frac{1}{1-K}\left(-1-\frac{d}{dc}\left\{\int_0^1[1-\Phi(\frac{T-\sigma\Phi^{-1}(p)}{\sqrt{1-\sigma^2}})]dp-\int_0^c [1-\Phi(\frac{T-\sigma\Phi^{-1}(p)}{\sqrt{1-\sigma^2}})]dp\right\}\right)$$

$$=\frac{1}{1-K}\left(-1+\frac{d}{dc}\int_0^c [1-\Phi(\frac{T-\sigma\Phi^{-1}(p)}{\sqrt{1-\sigma^2}})]dp\right)$$

$$=\frac{1}{1-K}\left(-1+[1-\Phi(\frac{T-\sigma\Phi^{-1}(c)}{\sqrt{1-\sigma^2}})]\right)$$

$$=\frac{-\Phi(\frac{T-\sigma\Phi^{-1}(c)}{\sqrt{1-\sigma^2}})}{1-K}$$

Note that $\int_0^1[1-\Phi(\frac{T-\sigma\Phi^{-1}(p)}{\sqrt{1-\sigma^2}})]dp$ is independent of *c*, and hence the derivative of this expression is 0.

Now we can express AUC as

$$AUC=\int_0^1 sens(c)\ d(1-spec(c))$$

$$=\int_1^0\left\{\int_c^1 [1-\Phi(\frac{T-\sigma\Phi^{-1}(p)}{\sqrt{1-\sigma^2}})]dp/K\times\left[-\Phi(\frac{T-\sigma\Phi^{-1}(c)}{\sqrt{1-\sigma^2}})/(1-K)\right]\right\}dc$$

$$=\int_0^1\left\{\int_c^1 [1-\Phi(\frac{T-\sigma\Phi^{-1}(p)}{\sqrt{1-\sigma^2}})]dp/K\times\left[\Phi(\frac{T-\sigma\Phi^{-1}(c)}{\sqrt{1-\sigma^2}})/(1-K)\right]\right\}dc$$

Note that when *c*=1, 1-specificity=0 and when *c*=0, 1-specificty=1, hence the change of integration limits on the 2nd line.

References:

1. Pepe MS (2003) The statistical evaluation of medical tests for classification and prediction. Oxford: Oxford University Press.
